# Targeted Amino Acid Substitutions in a *Trichoderma* Peptaibol Confer Activity against Fungal Plant Pathogens and Protect Host Tissues from *Botrytis cinerea* Infection

**DOI:** 10.3390/ijms21207521

**Published:** 2020-10-12

**Authors:** Marta De Zotti, Luca Sella, Angela Bolzonello, Laura Gabbatore, Cristina Peggion, Alessandro Bortolotto, Ibrahim Elmaghraby, Silvio Tundo, Francesco Favaron

**Affiliations:** 1 Department of Chemistry, University of Padova, Via Marzolo 1, 35131 Padova, Italy; laura.gabbatore@unipd.it (L.G.); cristina.peggion@unipd.it (C.P.); alessandro.bortolotto@unipd.it (A.B.); 2 Department of Land, Environment, Agriculture and Forestry, University of Padova, Viale dell’Università 16, 35020 Legnaro (Padova), Italy; luca.sella@unipd.it (L.S.); angela.bolzonello@studenti.unipd.it (A.B.); ibrahim_elmaghraby@yahoo.com (I.E.); silvio.tundo@unipd.it (S.T.); francesco.favaron@unipd.it (F.F.); 3 Agricultural Research Center, Central Laboratory of Organic Agriculture 9, Cairo Univ. St., Giza 12619, Egypt

**Keywords:** peptaibol, *Trichoderma*, green chemistry, bioactive peptides, antifungal activity, *Botrytis cinerea*

## Abstract

Fungal species belonging to the *Trichoderma* genus are commonly used as biocontrol agents against several crop pathogens. Among their secondary metabolites, peptaibols are helical, antimicrobial peptides, which are structurally stable even under extreme pH and temperature conditions. The promise of peptaibols as agrochemicals is, however, hampered by poor water solubility, which inhibits efficient delivery for practical use in crop protection. Using a versatile synthetic strategy, based on green chemistry procedures, we produced water-soluble analogs of the short-length peptaibol trichogin. Although natural trichogin was inactive against the tested fungal plant pathogens (*Botrytis cinerea*, *Bipolaris sorokiniana*, *Fusarium graminearum*, and *Penicillium expansum*), three analogs completely inhibited fungal growth at low micromolar concentrations. The most effective peptides significantly reduced disease symptoms by *B. cinerea* on common bean and grapevine leaves and ripe grape berries without visible phytotoxic effects. An in-depth conformational analysis featuring a 3D-structure–activity relationship study indicated that the relative spatial position of cationic residues is crucial for increasing peptide fungicidal activity.

## 1. Introduction

In the last decade, European Union (EU) policies have actively promoted research to reduce reliance on synthetic plant protection products (PPPs) for crop safeguarding in agricultural systems. Growing needs to implement integrated crop protection approaches demand novel alternatives for plant disease and pest control (EU Directive 2009/128/CE). The escalation of organisms that are resistant to PPPs is reducing the efficacy of several active substances. Besides, increasing restrictions limited the number of products available on the market (Regulation (EC) 1107/2009).

In this framework, integrated disease management exploits the so-called biocontrol agents, namely fungi and other microorganisms that can regulate the life cycles of phytopathogenic microorganisms. Fungi of *Trichoderma spp.* are among the most widely used and effective biocontrol agents [1,2]. *Trichoderma* fungi exert their antagonist action by nutrient competition, mycoparasitism through the secretion of degrading enzymes, and antibiosis by the production of secondary metabolites [3]. The major obstacle to the widespread use of *Trichoderma* spp. for controlling plant diseases is due to population vitality contingent upon environmental conditions [4]. The effectiveness of *Trichoderma* preparations is variable and sometimes unreliable under open-field conditions, such that use is mostly restricted against soil or seed-borne plant pathogens [1,5].

Natural products of plant or microbial origin have been pursued as starting points for the development of safer antimicrobial agents [6]. For plant protection, effective fungicides have been designed and developed starting from natural molecules, e.g., phenylquinoxalins, phenylpyrrole, and strobilurin-based fungicides [7,8]. *Trichoderma* spp. are an important source of secondary metabolites [9,10]. Many of those metabolites possess antifungal activity and have potential applications in crop protection [11]. The substitution of commercially available *Trichoderma* formulations with target-specific secondary metabolites for controlling phytopathogens was proposed as a tool for a next-generation fungicide era [10]. Many secondary metabolites produced by *Trichoderma* are toxic against microorganisms [10] and can act synergistically with enzymes to degrade the cell wall of parasitized fungi [12].

Among *Trichoderma* secondary metabolites, peptide antibiotics are the most abundant category, and those of the *peptaibol* family [13] constitute the largest group [14,15]. Peptaibols are 5–20 residue-long peptides, nonribosomally synthesized by fungi. The name *peptaibol* stems from two peculiar features of their primary structure: The presence of several noncoded, natural *Aib* (α-aminoisobutyric acid) residues and a C-terminal 2-aminoalcohol. The most studied and simplest of C^α^-tetrasubstituted α-amino acid residues, Aib is responsible for the remarkable resistance of peptaibols toward the action of proteolytic enzymes [16]. Aib is also a well-known helix inducer residue [17]. Consequently, even short peptaibols possess well-defined helical structures and stability at extreme pH values and high temperatures. Long-length peptaibols, such as the well-known alamethicin, assemble into helical bundles, which form channels in phospholipid membranes [18,19] by a mechanism generally associated with toxicity against microorganisms.

Few peptaibols have been assayed against plant pathogens. For example, the long-chain trichokonins from *Trichoderma pseudokoningi* have been characterized as programmed cell-death inducers against *Fusarium oxysporum* and *Botrytis cinerea* [20,21]. Trichokonins have enhanced defense responses in plants, permitting control of resistant phytopathogens, such as Gram-negative bacteria [22].

Despite promising features, limitations reduce the practical application of extracted peptaibols. The biosynthesis of peptaibols in nature is influenced by environmental factors and the presence of the antagonized species [23], conditions hard to reproduce in vitro [24]. The purification of peptaibols is not trivial, because they are secreted with other secondary metabolites in microheterogeneous mixtures. Naturally occurring peptaibols contain few polar residues in their sequence. Hence, they are, in general, poorly soluble in water. This feature hampers their efficient delivery to the target and often results in ineffective plant protection. Such drawbacks prompted us to produce water-soluble peptaibol analogs for the control of fungal plant pathogens. The use of purified compounds instead of complex mixtures of congeners may minimize environmental contaminants, remove variability, and enhance reproducibility.

For our biorational approach, we chose the short-length, natural peptaibol trichogin GA IV (hereafter called trichogin) [25]. Trichogin possesses antimicrobial activity [26] and a well-developed helical structure [27], but poor solubility in water. In the trichogin sequence (Oct-Aib-Gly-Leu-Aib-Gly-Gly-Leu-Aib-Gly-Ile-Lol, Table 1), Oct is 1-octanoyl and Lol is the 2-aminoalcohol leucinol. The presence of a lipophilic acyl chain at the N-terminus makes trichogin a *lipo*peptaibol [28] and has been associated with inhibition of plant-pathogenic bacteria and fungi [29]. This helical, mostly hydrophobic, 11-residue peptaibol is naturally produced by *T. longibrachiatum*, and its activity has been assayed in various biological systems [26,30,31]. In this manuscript, we describe the synthesis, conformational analysis, proteolytic resistance, and fungicidal activity of water-soluble, cationic analogs of trichogin (Table 1). Some of the analogs synthesized and tested in the present work were chosen in light of the promising results obtained for them in previous studies involving human bacterial pathogens [30,32]. Cationic peptides have been previously found to be effective against fungal plant pathogens [33,34]. In the present work, the poor reactivity of Aib residues has been successfully overcome using a green [35] and cost-effective synthetic strategy to produce trichogin analogs.

The fungicidal activity was tested against fungal plant pathogens belonging to different ascomycete classes (*Botrytis cinerea*, *Bipolaris sorokiniana, Fusarium graminearum,* and *Penicillium expansum*). A more in-depth investigation with the most active compounds was performed on *Botrytis cinerea*, a highly polyphagous necrotrophic pathogen among the most dangerous pathogens on crops and fruits before and after harvesting worldwide [36]. The selected fungi are important pathogens that cause respectively foliar spot blotch and black point on grain (*B. sorokiniana*), the blight of spike in cereals (*F. graminearum*), and rot on apple (*P. expansum*). They are also producers of phytotoxins and mycotoxins in plants [37,38,39].

## 2. Results

### 2.1. Peptide Synthesis and Characterization

**Figure 1:**
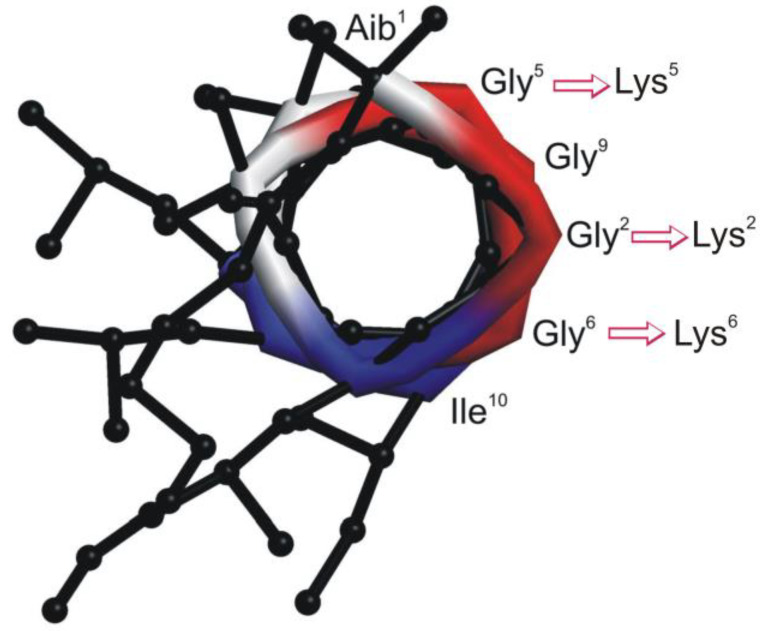
Helical structure of the native peptaibol trichogin (view along the helical axis) [27]. Substitutions of Lys for Gly were made on the less hydrophilic face.

To create trichogin analogs with improved water solubility, modifications were made on the less hydrophilic face of the amphiphilic, helical conformation (Figure 1).

Building on previous studies [32], peptide amphiphilicity was improved by replacing one or more Gly residues by Lys (peptides **1–5** and **7**). To identify the minimal peptide length needed for bioactivity, we also prepared shorter analogs (peptides **4c**, **4c1** and **4c2**). A more rigid helical structure was obtained by replacing Gly^6^ with an Aib residue (peptide **6**). Finally, we tested the effect of perturbing the hydrophobic face of the helix by replacing Aib at position 8 with the cationic, C^α^-tetrasubstituted 4-aminopiperidine-4-carboxylic acid (Api) residue (peptide **8**). To better visualize the effect of sequence modifications on helix amphiphilicity, we built structural models for all analogs (Figures S1–S4, Supplementary Materials).

In view of the possible industrial production of the analogs as plant protection products, the relatively expensive C-terminal -Lol moiety was replaced with a -Leu-NH_2_ (leucine amide) residue in peptide **4r** on Rink Amide resin.

Peptides were produced by manual and semiautomatic solid-phase peptide synthesis (SPPS). To reduce the impact on the environment of the synthesis, a limited excess of reagents was employed in the coupling steps (1.5–2.5 equivalents of coupling reagents instead of the standard 4 equivalents-1 equivalent corresponds to the loading of the resin). Moreover, all steps were performed in the ecofriendly solvent mixture of dimethylsulfoxide/ethyl acetate 1:9 instead of the hazardous solvent N,N-dimethylformamide (DMF) [40]. We exploited ethyl cyano(hydroxyimino) acetate (Oxyma pure) and diisopropylcarbodiimide (DIC), classified by the ACS Green Chemistry Institute^®^ Pharmaceutical Roundtable as two of the greenest coupling reagents [41], instead of the commonly employed O-benzotriazol-1-yl-1,1,3,3 tetramethyluronium hexafluorophosphate (HBTU)/1-hydroxy-1,2,3-benzotriazole (HOBt) mixture or HATU [2-(1H-7-aza-1,2,3-benzotriazol-1-yl)-1,1,3,3-tetramethyl uronium hexafluorophosphate]. The strategy reduced the environmental impact and synthetic costs by more than 70% compared to our previous methods [32] based on standard procedure. By adopting a manual/semiautomatic synthetic approach, the amount of solvents and reagents were reduced relative to those used in automated synthesis. The steric hindrance of Aib makes it poorly reactive, to the point that standard procedures employ repeated couplings. Nonetheless, we managed to successfully carry out the synthesis with very good crude purities performing double couplings only to link Leu (or nOct) onto Aib. We repeated the synthesis of all analogs several times and unequivocally established that Aib can be successfully inserted onto a growing peptide chain up to ten residues long by a single coupling with 2.5 equivalents. All crude peptides were obtained with a degree of purity higher than 85% and purified to 95–99% by semiautomatic medium-pressure chromatography using only one liter of eluants (mostly water, thanks to the water solubility of the analogs) per gram of crude peptide. The post-purification yield for all syntheses was very good (above 70%). To further improve the synthesis, we examined a previously reported chemoenzymatic approach [42] for making Aib peptides [43], but failed to obtain the targeted sequences. All peptides were fully characterized (see Supplementary Materials) and their water solubility proven up to 0.01 M (the highest concentration tested).

### 2.2. Proteolytic Stability Assays

Trichogin analogs were shown by HPLC to be resistant to enzymatic degradation by proteolytic enzymes such as pronase E (a mixture of different proteases) and trypsin. The shortest sequences were not analyzed. All tested peptides were completely stable in the presence of the enzymes after 24 h, except of peptide **1**, which was stable against pronase after 27 h, but digested by trypsin in less than 15 min. The digestion of peptide **1** by trypsin can be due to the known conformational flexibility of the native trichogin at the level of Gly^5^-Gly^6^ kink [27]. Such flexibility may indeed give better access to the basic residue Lys^5^ in peptide **1** and facilitate the bond cleavage by trypsin.

### 2.3. Activity of Peptides against B. cinerea

The eight full-length peptides (Table 1) were assayed for their ability to inhibit the growth of *B. cinerea*. To this aim, conidia were incubated in microtiter wells supplied with the peptides at a concentration of 15 µM in the presence of the resazurin vital dye. During the following seven days, the plates were monitored every 12 h for fungal growth, as revealed by the color change of the resazurin dye. Without treatment, the fungus began to grow in the first 12 h. Similarly, fungal growth was observed after 12 h in the presence of peptide **3** and trichogin. A delayed growth was observed in wells containing **1**, **2**, **6**, and **7**. No growth occurred in wells containing conidia treated with **4**, **5**, or **8** (Table 2).

The analogs of **4** (**4r**, **4c**, **4c1**, and **4c2**), with shorter and modified amino acid sequences, also exhibited activity against *B. cinerea* and were effective at 15 µM, inhibiting mycelial growth (Table 2).

**Figure 2:**
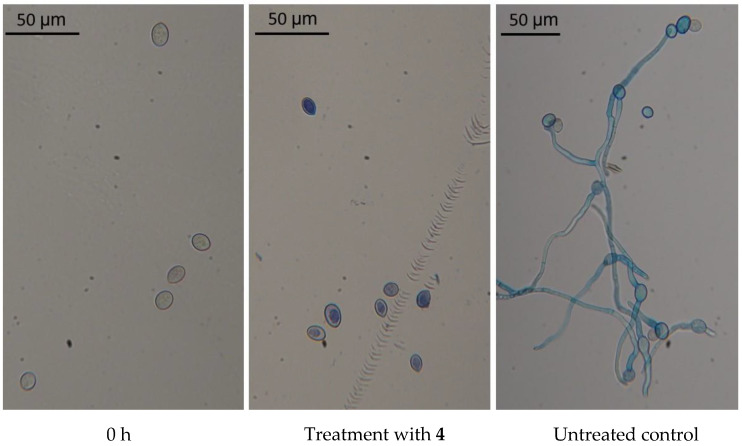
Evan’s blue staining of *B. cinerea*. Conidia of *B. cinerea* were stained at 0 h and after 24 h from treatment with **4** at 15 µM. Untreated conidia (control) were stained after 24 h.

A cell viability assay was performed by incubating *B. cinerea* conidia for 24 h with **4**, which was one of the three most active peptides. The microscopic examination showed that conidia did not germinate. Evans blue vital dye labeled the protoplast of conidia, which appeared reduced in size and detached from the cell wall. In the untreated control, conidia germinated and produced elongated hyphae (Figure 2).

Conidial suspensions treated with **4** at 15 µM were rinsed with distilled water and plated on potato dextrose agar (PDA). No fungal growth was observed, revealing the fungicidal activity of the peptide at that concentration.

### 2.4. Comparison of Peptide Fungicidal Activity against Fungal Plant Pathogens

**Figure 3:**
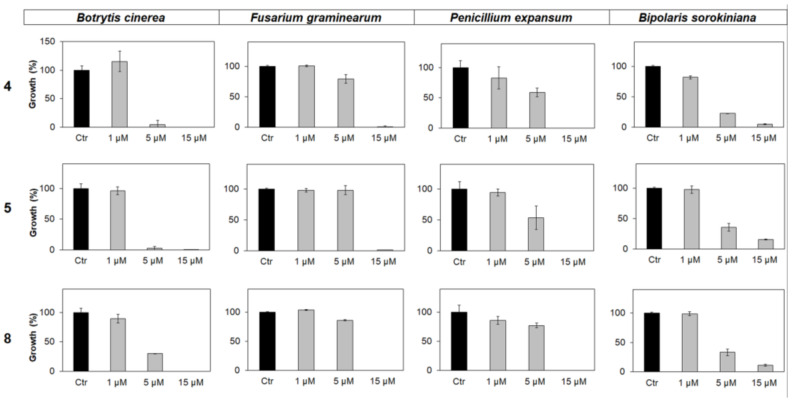
In vitro antimicrobial activity of **4**, **5**, and **8** against the fungal pathogens (from **left** to **right**) *B. cinerea*, *F. graminearum, P. expansum,* and *B. sorokiniana*. Peptides were supplied at 1, 5, or 15 µM concentration. After 96 h of incubation, the fungal growth was estimated by reading the absorbance at 450 nm, and it was expressed as a percentage of the maximum values measured in control samples containing the corresponding fungal species not treated with the peptides. Each data point is the mean of 3 independent replicates ± standard error (SE).

Further assays allowed us to obtain the minimal inhibitory concentration (MIC) values for the most active peptides (**4**, **5**, and **8**) against *B. cinerea* and other ascomycete fungal pathogens (*B. sorokiniana*, *F. graminearum*, and *P. expansum*). Peptides **4** and **5** displayed MIC values between 1 and 5 µM against *B. cinerea*. At the maximum concentration tested (15 µM), all three peptides completely inhibited the growth of *B. cinerea*, *F. graminearum*, and *P. expansum* and showed a strong growth inhibition effect against *B. sorokiniana* (Figure 3).

### 2.5. Peptide Protection of Plant Tissues from B. cinerea Infection

**Figure 4:**
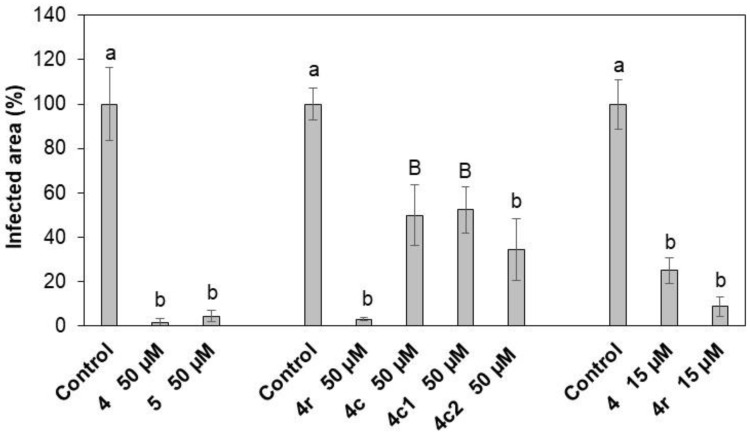
Symptomatic area of common bean leaves treated with **4**, **4r**, and **5**, and the shorter analogs **4c**, **4c1**, and **4c2** at 50 or 15 µM and inoculated with *B. cinerea* conidia. Each treatment was compared to the respective control. The symptomatic leaf area was measured 7 days post-inoculation (dpi) and is expressed as a percentage compared to the control (treated with water). Bars indicate standard error. At least three independent infection sessions were performed. Data were statistically analyzed by one-way Anova Bonferroni–Holm. Different letters indicate significant differences at *p* < 0.01 (lowercase letters) or *p* < 0.05 (uppercase letters).

**Figure 5:**
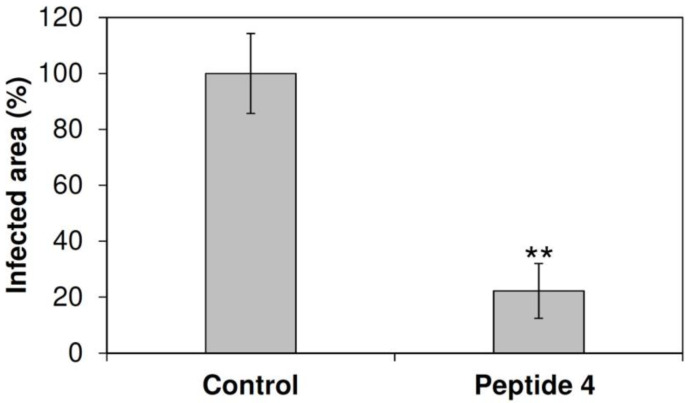
Symptomatic area of grapevine leaves cv. Glera treated with **4** at 50 µM and inoculated with *B. cinerea* conidia. The symptomatic leaf area was measured 7 days post-inoculation (dpi) and is expressed as a percentage compared to the control (treated with water). Bars indicate standard error. Three independent infection experiments were performed. Data were statistically analyzed by the Student’s *t*-test. Each treatment was compared to the respective control. ** indicates significant differences at *p* < 0.01.

Peptides **4** and **5** were identified as the most effective against *B. cinerea* in vitro and used to treat common bean leaves to study efficacy against *B. cinerea* infection. At 50 μM, both peptides reduced symptoms, diminishing the infected leaf area by >95% (Figure 4; Figure S5, Supplementary Materials). At 15 μM, peptide **4** reduced the *B. cinerea* average lesion area by about 75% (Figure 4; Figure S5, Supplementary Materials). Moreover, peptide **4** protected grapevine leaves and berries from *B. cinerea* infection. At 50 μM, **4** reduced the infected leaf area by about 70% (Figure 5) and disease on berries by about 45% compared to the untreated control (Table 3; Figure S6, Supplementary Materials). Interestingly, the peptide treatment resulted in a threefold increase in the number of asymptomatic berries (Table 3). No phytotoxic effects were noticed on either leaves or berries after treatment with the peptides (data not shown).

### 2.6. Protection of Common Bean Leaves from B. cinerea Infection by Peptide 4 and Modified Analogs

We tested the efficacy in vivo of the shorter and modified analogs of **4** to protect common bean leaves from *B. cinerea* infection. Peptide **4r** (modified at the C-terminus) was the most effective at 50 μM, and reduced the symptomatic leaf area by about 95% (Figure 4). At the same concentration, the shorter versions **4c2**, **4c**, and **4c1** (spanning the N- or C-terminal half of the sequence) were less effective in protecting bean leaves, with a reduction in the infected leaf area by about 70%, 55%, and 55%, respectively (Figure 4). In addition, **4r** was more effective than **4** at 15 µM, decreasing symptomatic leaf area by about 90% (Figure 4).

### 2.7. Conformational Studies

Peptaibols in general—and trichogin in particular—feature stable and well-defined helical conformations. To make sure our substitutions did not alter the native 3D structure, we studied the conformational preferences of our trichogin analogs in solution by means of circular dichroism (CD) [44] and 2D-NMR. The helical structure proved important for peptide bioactivity. Helical stability was also investigated in the presence of fungi. To the best of our knowledge, peptide conformational analysis in vitro in the presence of phytopathogenic fungi has not yet been described in the literature [45].

#### 2.7.1. Circular Dichroism Spectroscopy

**Figure 6:**
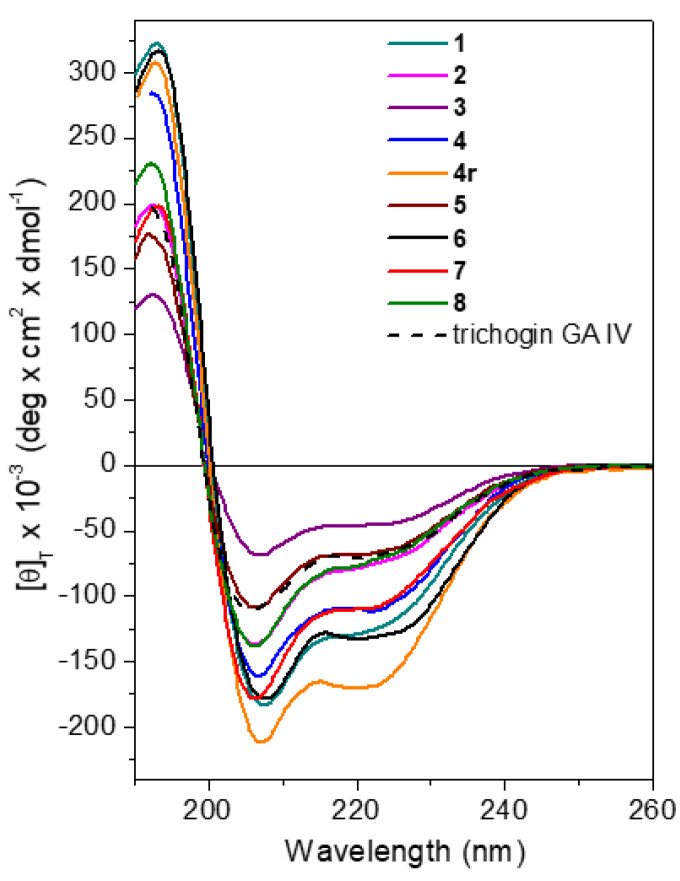
CD profiles recorded for trichogin analogs in SDS 100 mM aqueous solution (peptide concentration: 10^−4^ M). The spectrum of native peptide trichogin GA IV is shown for comparison.

Circular dichroism (CD) spectroscopy is a powerful technique for obtaining information regarding the conformation assumed in solution by peptides. Measurements were performed at room temperature using aqueous solutions of peptide at 10^−4^ M. For the longer trichogin analogs, which may act as membrane-active peptides, CD analysis was performed in the presence of 100 mM sodium dodecyl sulfate (SDS) micelles in water. All peptides displayed spectral signatures typical of right-handed helical conformations, with negative maxima centered at about 208 and 222 nm (Figure 6).

Aib-containing peptides are known to adopt either 3_10_- [46,47] or α-helices. The two helical types give rise to dichroic curves in which these two negative maxima have different relative intensities [46,47]. In the α-helix, the band at 208 nm and that at 222 nm have almost the same intensity. In the 3_10_-helix, the latter has a much lower intensity than the former and tends to blue shift. The relative amount of the two helical types can be estimated from the ratio *R* between the molar ellipticities at 222 and 208 nm (*R* = [θ]_222_/[θ]_208_), which are respectively <0.4 and 1 for the 3_10_- and α-helices. All trichogin analogs exhibited helical CD profiles in the membrane-mimicking environment. A ratio *R* of about 0.7 was found for all peptides. We conclude that the right-handed, mixed 3_10_-/α-helical conformation typically adopted by the native peptide trichogin GA IV is preserved in the Lys-containing counterparts. Peptides **6** and **8**, which contain an additional Aib and the C^α^-tetrasubstituted Api residue, respectively, exhibited a larger helical content compared to the other analogs. Moreover, the CD spectrum of **4r** possessed stronger negative maxima, likely due to the C-terminal primary amide offering additional potential for hydrogen bonding compared to the corresponding Lol-analogs (e.g., **4**).

The 3D structure of shorter analogs **4c**, **4c1**, and **4c2** were investigated in water (Figure S7, Supplementary Materials). A negative dichroic band falling below 200 nm is associated with the presence of an unordered 3D structure [48], and longer wavelengths (205–208 nm) are found for helices [48]. The values found for **4c**, **4c1**, and **4c2** (202, 202, and 203 nm, respectively) were indicative of the presence of a helical structure, but rather red-shifted compared to those of full-length analogs (for which the band falls at about 208 nm, Figure 6). Such a position shift suggests a loss in conformational stability that might contribute to the loss of bioactivity registered for the shorter analogs.

**Figure 7:**
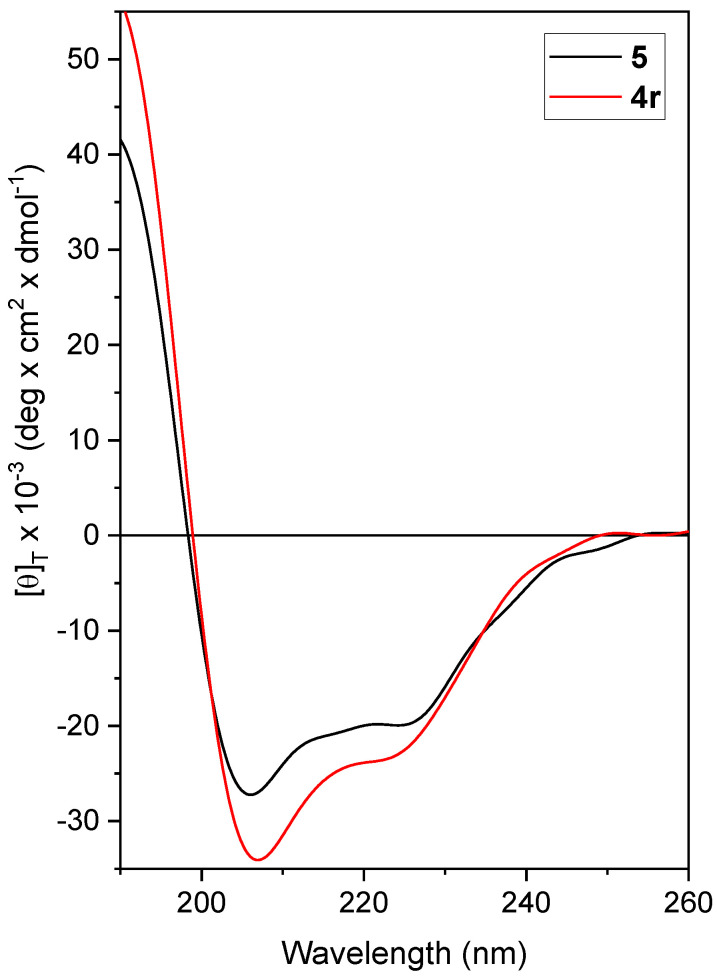
CD spectra acquired for **4r** and **5** in vitro, in the presence of fungal spores in phosphate buffer pH 7, 25 °C. Peptide concentration: 50 µM.

Finally, we set-up the experimental conditions and performed CD analysis on solutions of fungal spores treated with each of the most effective peptides. All peptides displayed a stable helical conformation in the presence of fungal spores. The CD profiles obtained for the leading peptides **4r** and **5** are reported in Figure 7.

#### 2.7.2. D NMR Spectroscopy

The conformational preferences of the short analogs **4c** and **4c1** in water were also studied by 2D NMR spectroscopy (p. S36–S37 and S38, respectively, Supplementary Materials). The region of the ROESY (rotating frame nuclear Overhauser effect spectroscopy) spectrum in which NH-NH correlations are normally found featured all sequential cross-peaks, apart from those hidden under the diagonal. The presence of a helical conformation was confirmed. Structural flexibility was indicated by the absence of long-range cross-peaks in the *fingerprint* region of the short analogs (as an example, see p. S37 Supplementary Materials).

The presence of a NH-NH ROESY correlation between Leu^11^ αNH and the C-terminal capping amide moiety (-NH_2_) for **4c1** (p. S28, Supplementary Materials) indicated the onset of an additional, stabilizing H-bond, involving the C-terminal primary amide moiety. We can hypothesize a slightly higher helical stability for **4c1** and **4c2** than **4c** (featuring a C-terminal Lol).

**Figure 8:**
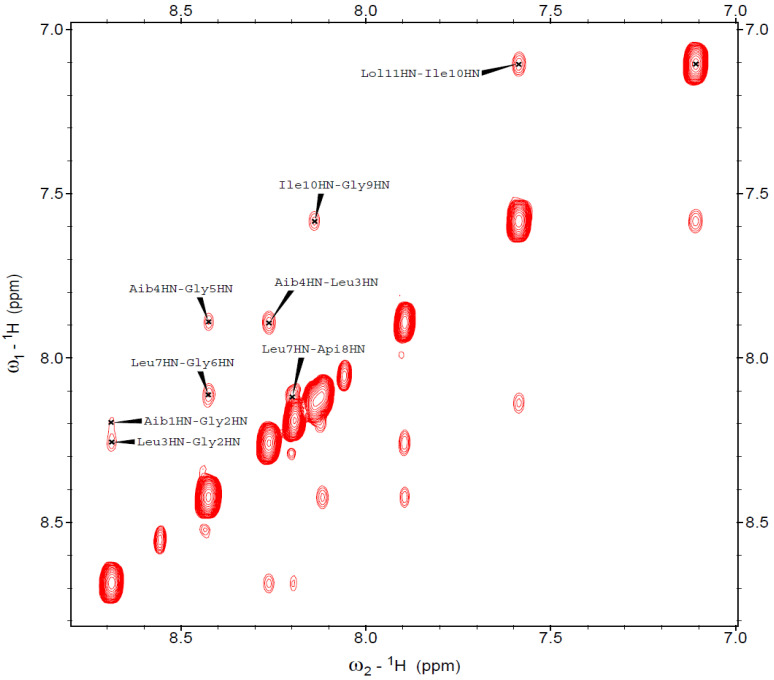
NH amide region of the NOESY spectrum acquired for peptide **8** (600 MHz, 100 mM SDS-*d_25_*, H_2_O/D_2_O 9:1, pH: 4.5, 313 K). Peptide concentration: 1.66 mM.

**Figure 9:**
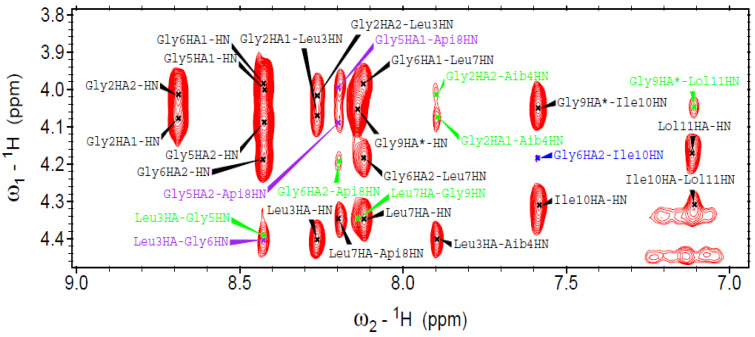
*Fingerprint* region of the NOESY spectrum acquired for peptide **8** (600 MHz, 100 mM SDS-*d_25_*, H_2_O/D_2_O 9:1, pH 4.5, 313 K). Peptide concentration: 1.66 mM. The medium- and long-range interactions are shown in different colors (*i*→*i* + 3, **purple**; *i*→*i* + 2, **green**; *i*→*i* + 4, **blue**).

We also performed a complementary 3D-structural characterization by 2D NMR of selected full-length peptide analogs in the presence of micelles (100 mM SDS-d_12_ in H_2_O/D_2_O 9:1). For example, peptide **8** ([Api^8^]-trichogin) was helical and exhibited characteristic long-range magnetization transfer in the NOESY spectrum (Figure 8 and Figure 9). All sequential NH_(*i)*_→NH_(*i+1)*_ cross-peaks were detected, apart from that of Gly^5^-Gly^6^ that fell under the diagonal. The *fingerprint* region (Figure 9) featured medium- and long-range cross-peaks (C^α^H_(*i*)_→NH_(*i*+2)_ and C^α^H_(*i*)_→NH_(*i+*4)_, diagnostic of 3_10_- and α-helices, respectively). Overall, we confirm that peptide **8** adopted a well-developed, mixed 3_10_-/α-helix under the investigated experimental conditions. It is evident that Aib-to-Api (both C^α^-tetrasubstituted residues) substitution at position 8 does not alter the native 3D structure.

## 3. Discussion

Water-soluble trichogin analogs proved active against the ubiquitous fungal plant pathogen *B. cinerea*. Although trichogin was found to be completely inactive against this fungus, seven out of the eight full-length analogs resulted in different levels of inhibition of fungal growth. Peptide **4**—used for a deeper analysis against fungal spores—was unambiguously found to kill spores of *B. cinerea*. Peptides **4**, **5**, and **8** completely inhibited the growth of *B. cinerea* in vitro with MIC value (Figure 3) in the range 1–5 µM. *F. graminearum* and *P. expansum* also resulted susceptible to peptides **4**, **5**, and **8**, with MIC in the range 5–15 µM.

Peptaibols are membrane-active peptides that exert their activity by forming aggregates on biological membranes [13]. Their efficacy against fungal pathogens may depend on the size, shape, and structural characteristics of conidia. The limited number of fungal species analyzed in this work cannot allow a conclusive demonstration of the relationship between the size and shape of conidia and sensitivity to peptides. Nonetheless, in comparison to the other tested fungi, *B. sorokiniana* has much larger spores [49] and our analogs did not completely arrest its growth at the highest concentration tested (15 µM, Figure 3).

Peptides **4** and **5** were identified as the most effective in in vitro experiments and tested in vivo. Effectiveness was confirmed for both peptides on common bean leaves inoculated with *B. cinerea*, with a significant decrease in disease symptoms at 15 µM and almost complete annulment at 50 µM concentration (commercially available pesticide active substance fenhexamid [MW 302 g/mol] is applied at 0.5 g/L (1.65 mM) concentration to control *B. cynerea* [50]). The protection provided by peptide **4** against *B. cinerea* is neither plant- nor organ-specific, as confirmed by infection experiments of grapevine leaves and berries. The results on berries are particularly interesting as they highlight the ability of these molecules to protect ripe fruits that are highly susceptible to infection by *B. cinerea.*

Remarkable conformational stability and persistence accompany the effectiveness of our peptaibols as antifungal molecules. We found that most full-length trichogin analogs are resistant to proteolytic degradation for more than 24 h. By CD (data not shown), we confirmed that their 3D structure is stable under critical pH (pH = 1) and temperature (up to 120 °C in DMSO) conditions. Overall, the data suggest an excellent persistence under open-field conditions.

Aib-to-Api substitution (peptide **8**) did not change the structural stability but perturbed the amphipathicity of the helix structure of trichogin by introducing a positively charged residue in its hydrophobic face. Such modifications were previously reported to enhance peptide cytotoxic activity against tumor cell lines [31]. The high fungicidal activity of peptide **8**, that bears Api at position 8, is probably due to this unique structural feature.

We exploited Gly-to-Lys substitutions both to confer water solubility to trichogin analogs and to enhance their fungicidal activity. Nonetheless, the presence of more than one Lys residue in the sequence seems to not be associated with dramatic changes in peptide activity against *B. cinerea*. On the other hand, when two Lys residues are present in the sequence, Lys relative position does seem to play an essential role in peptide performance. A summary of peptide antifungal effectiveness against *B. cinerea versus* Lys positions is reported in Table 4. The well-developed helical 3D structure adopted by all the synthesized analogs allowed us to draw a 3D-structure–activity relationship for the full-length analogs **1–7**. Lys^2^ and Lys^6^ in peptide **7** are one helix-turn away, so spatially close. Spatial proximity between the two—positively charged—Lys residues can cause electrostatic repulsions that, in turn, might influence peptide performance against the target pathogen(s). That might explain why peptide **7** is less active than peptide **5** (with Lys at position 6 only). The same holds true for peptide **1**, where Lys at position 5 is also close to Lys^2^. In the case of peptide **4**, such electrostatic repulsion between Lys^5^ and Lys^6^ can be avoided because the two residues, although close in sequence, point toward different directions. Peptide **4** and **5** display the same efficacy in controlling the development of *B. cinerea* symptoms *in planta*. Thus, Lys at position 6 is needed to increase peptide efficacy against *B. cinerea* (with no other Lys residues spatially close).

The modified analogs of peptide **4** were as active as **4** in vitro against *B. cinerea* but showed different efficacy in limiting disease symptoms on common bean leaves. Peptide **4r**, modified at the C-terminus, was as effective as **4** and more effective than the shorter analogs **4c2**, **4c**, and **4c1**. This result could depend on the loss in conformation stability observed for shorter peptides in CD experiments.

In conclusion, by using an ecofriendly, versatile SPPS strategy, we obtained several water-soluble analogs of the naturally occurring peptaibol trichogin GA IV with high yield and crude purities. Two leading peptaibol analogs (**4** and **5**) are significantly active in vitro against the fungus *B. cinerea*, the causal agent of gray mold, and other ascomycete fungal plant pathogens (*B. sorokiniana*, *F. graminearum*, and *P. expansum*). Peptide **4** was also successfully used for the protection of leaves and grape berries, proving interesting for the development of an effective product against gray mold under open-field conditions. Active peptides maintain their helical structure while exerting antifungal activity. Further investigations are planned to understand whether our peptaibol analogs can also enhance defense responses in plants. We also plan to make negatively charged counterparts of peptide **4** to differentiate the solubilizing and cation effects.

## 4. Materials and Methods

### 4.1. Peptide Synthesis

Peptides were obtained by manual or semiautomatic (Biotage MultiSynTech) SPPS on a 0.3–1 mmol scale. This method allowed us to reduce the amount of solvent for each synthesis, as well as the excess of coupling reagents compared to a fully automatic SPPS (automated synthesis requires dead volumes, namely at least one equivalent of all Fmoc-amino acids and coupling reagents more than manual synthesis. Several steps of needle cleaning are also needed, with consequent waste of solvent). We used either the rink-amide resin or the 2-chlorotrityl resin preloaded with the 2-aminoalcohol L-leucinol, both commercially available. The protocol makes use of the standard fluorenylmethyloxycarbonyl (Fmoc-) protecting group, removed by treatment with 20% piperidine in DMF (2 mL for 0.75 g of resin). The ε-NH_2_ groups on the side-chain of Lys residues were protected by *tert*-butyloxycarbonyl (Boc) groups, which can be removed in acidic conditions, such as a 3M HCl solution in methanol (or trifluoroacetic acid, TFA).

The activation of the carboxyl group of the incoming amino acid was achieved using Oxyma pure and DIC [35,51] (Fmoc-AA/Oxyma/DIC 1:1:1) using 1.5 (for Gly and Ile) or 2.5 equivalents with respect to the loading of the resin, instead of the standard 4 equivalents. The standard SPPS procedure outline includes double coupling for each and every step involving Aib residues. The poor reactivity of Aib due to the steric hindrance of the gem dimethyl groups, however, mostly hampers the nucleophilic attack by Aib αNH_2_ onto the incoming, activated Fmoc-Leu-OH/*n*Octanoic acid. Thus, only that step really needs to be repeated with fresh reagents. Therefore, we performed double couplings only to link Fmoc-Leu-OH (or *n*Oct) onto Aib. We repeated the synthesis of all analogs several times and unequivocally established that Fmoc-Aib-OH can be successfully inserted by a single coupling with 2.5 equivalents. All washings and coupling steps were performed in dimethylsulfoxide/ethyl acetate 1:9 [40]. Peptides were released from the resin by acidic treatment [52]. The procedure must be repeated at least three times to obtain complete peptide release, and then additional treatment with HCl 3M in methanol is needed to remove the *tert*Butyloxycarbonyl (Boc) side-chain protection (either on Lys or Api). Release from Rink-amide resin was achieved by treatment with a mixture of 95% TFA, 2.5% water, and 2.5% triisopropylsilane (2 mL for 0.75 g of resin). The collected solution was concentrated to dryness and the obtained solid (or oily) precipitate was washed twice with diethyl ether. Conversely, peptides were released from 2-chlorotrityl resin by repeated treatments with a solution of 1,1,1,3,3,3-hexafluoroisopropanol 30% in dichloromethane (3 × 2 mL for 0.75 g of resin) lasting from one hour to overnight. Such mild acidic conditions do not cleave side-chain protections. Thus, Boc protecting groups were removed in solution by treatment with 3M hydrochloric acid in methanol (2 mL for 0.75 g of resin).

Peptides were obtained with very good crude purities (85–97%) and easily purified to 95–99% purity by medium-pressure liquid chromatography (Isolera Prime, Biotage, Uppsala (Sweden)). To purify one gram of crude peptide from 80% to >95% we used 500 mL of water milliQ and 150mL of acetonitrile. We used HCl instead of TFA (1 mL of HCl 37% per 1L of eluant). After peptide elution, we washed the column with ethanol and water (300 mL in total). Peptide characterization (reported in the Supplementary Materials) was obtained through high-resolution mass spectrometry (HRMS) analysis (electrospray ionization time-of-flight, ESI-TOF), analytical HPLC (used also to determine the degree of purity), and nuclear magnetic resonance (^1^H NMR). Yields obtained (after purification): always > 70%. Full characterizations for all peptides are reported in the Supplementary Materials (p. S7–S42).

### 4.2. Fungal Strains and Conidia Production

*Botrytis cinerea* strain PM10 [53], *Fusarium graminearum* strain PH1 [54], *Bipolaris sorokiniana* strain 62,608 DSMZ, and *Penicillium expansum* strain PVPD2016_3 were cultured at 25 °C on potato dextrose agar (PDA, Difco Laboratories, Detroit, MI, USA). Conidia were recovered from 15 days-old, completely colonized plates by gently scraping with a glass rod in the presence of 6 mL of sterile water supplemented with glycerol (10% *v*/*v*). For *F. graminearum*, spores were obtained by culturing the fungus in carboxymethyl cellulose (CMC)-containing medium, as reported in [55]. Conidia were filtered through sterilized gauze and counted using a hemocytometer.

### 4.3. In Vitro Assays

The antimicrobial activity of the peptide analogs synthesized in this work was initially evaluated against *B. cinerea* with the resazurin vital dye assay [54]. In light of the poor water solubility of native trichogin, we solubilized it in ethanol before diluting it in water to obtain a final ethanol concentration of 5% (*v*/*v*). Wells of microtiter plates were filled with 200 μL of potato dextrose broth (PDB; Difco Laboratories) at pH 6.9 supplemented with the peptides at 15 μM, previously resuspended in water, and with *B. cinerea* conidia at a final concentration of 5 × 10^5^ spores mL^−1^. Controls without peptides were also prepared. To each well, 68 µg mL^−1^ of resazurin dye (Sigma-Aldrich, Milano, Italy) were added. The microtiter plates were then incubated in the dark at 25 °C and monitored every 12 h for resazurin dye color change. The time at which control wells, without peptides added, turned into pink was fixed as time zero to record the delay of growth caused by the peptides. Each peptide was tested in three independent replicates.

The most active peptides were then assayed at 1, 5, and 15 μM against the fungal pathogens *B. sorokiniana, F. graminearum*, and *P. expansum*, as well as *B. cinerea*. Wells of microtiter plates were filled as reported above, supplemented with different concentrations of peptides, without the addition of resazurin, and incubated in the dark at 25 °C. After 96 h of incubation, the fungal biomass in the wells was estimated by reading the absorbance at 450 nm [56], and it was expressed as a percentage of the maximum values measured in control wells containing the corresponding fungal species not treated with the peptides. Each data point is the mean of 3 independent replicates. The minimal inhibitory concentration (MIC) values were also calculated.

### 4.4. Microscopy Analysis

A cell viability assay was performed by treating 5 × 10^5^ mL^−1^ conidia of *B. cinerea* suspended in PDB with peptide 4 at 15 µM. At 0 h and after 24 h, Evan’s Blue staining was performed to label fungal protoplasts of dead cells. Evan’s blue staining protocol was adapted from [57] and [58] to enhance the staining. Briefly, 100 µL of incubation mixture were concentrated by centrifugation at 3000× *g* for 10 min. Evan’s Blue dye was added to a final concentration of 0.05% (*w/v*), and the mixture was incubated for 15 min at room temperature. After incubation, fungal spores were abundantly washed with phosphate-buffered saline (PBS), centrifuged as above reported, visually analyzed for vitality by optical microscopy (Laborlux 12), and photographed (Sony 20.1 Megapixel AVCHD camera, Tokyo, Japan). Conidia suspended in PDB were used as a negative control.

Aliquots of the suspensions treated with peptide 4 for 24 h were abundantly rinsed with sterile distilled water, centrifuged, and plated on PDA to check conidia viability.

### 4.5. In Vivo Assays

The most effective peptides against *B. cinerea* in vitro were further assayed against *B. cinerea* infection of bean leaves and grapevine leaves and berries.

Leaves of common bean (*Phaseolus vulgaris*, Borlotto var. Teggia) were detached from 12 days-old plants grown in organic soil into a climatic chamber with a 14 h photoperiod and 22/20 °C day/night temperature. Leaves of a white grapevine variety (*Vitis vinifera*, cv. Glera) were collected from vine cuttings grown in the climatic chamber for about one month after potting. Grape berries (cv. Pinot blanc) were harvested at maturity (by mid-August) from a typical vineyard included in the municipality of Conegliano (North-East Italy). Ripe grape berries were detached from the bunch, surface-sterilized with 0.115% (*v/v*) sodium hypochlorite for 15 min, rinsed with sterile water, and separately arranged on a plastic tray (Figure S7, Supplementary Materials).

Treatments were performed by spraying the peptides at different concentrations on leaves and berries. Water spraying was performed as a negative control.

Leaves were arranged on moist towels in plastic trays and inoculated with a suspension of 2.5 × 10^6^ mL^−1^ conidia of *B. cinerea* in potato dextrose broth (PDB). Ten microliters of the conidia suspension were dropped on two or three points apart on the leaf surface of common bean and grapevine leaves, respectively. Berries were sprayed with *B. cinerea* conidia at a concentration of 1.85 × 10^6^ mL^−1^.

Inoculated leaves and berries were incubated in the dark at 25 °C in plastic bags to maintain humidity.

Seven days post-inoculation (dpi), the size of the symptomatic leaf area was measured from pictures taken with a Panasonic Lumix DMC-FZ2000 by using the graphic software “Fiji ImageJ” (GNU General Public License). For grape berries, symptoms were assessed at 4 dpi. As the *B. cinerea* infection produces a visible browning spot, each infected berry was indexed based on symptoms development using a Disease Index (DI) modified from [59]. Specifically, the DI ranged from 0 to 4, where 0 = healthy berries without lesion spots, 1 = infected berries with small spots covering <5% of the berry surface, 2 = infected berries with spots covering <25% of the berry surface, 3 = infected berries with spots covering >25% of the berry surface, and 4 = infected berries with spots covering >50% of the berry surface.

The experiment was repeated three times with leaves and four times with berries. Each treatment was performed with seven leaves of common bean and three leaves of the grapevine. Twenty grape berries were used for each treatment.

Data obtained in the infection experiments performed on common bean leaves were statistically analyzed by applying the one-way Anova Bonferroni–Holm test. The statistical analysis was performed within the groups of peptides tested at the same time compared to their respective control. Data obtained in the infection experiments performed on grapevine leaves and berries were statistically analyzed by applying the two-tailed Student’s *t*-test.

### 4.6. Conformational Analysis

CD was performed on a Jasco 1500 spectrometer using 1 mm-pathlength cells. Water and a solution of SDS 100 mM in water were used as solvents. Then, 16–32 scans were acquired at 25 °C between 190 and 280 nm. Peptide concentration: 0.1 mM. CD in fungi was performed on a sample prepared in 10 mM phosphate buffer at pH 7 incubated at 25 °C in the dark. Peptide final concentration: 50 µM. 2D NMR spectra were acquired at 313 K with a Bruker AVANCE DMX-600 instrument operating at a frequency of 600 MHz for ^1^H.

## 5. Patents

The Italian Patent N. 102018000006817 resulted from the work reported in this manuscript.

## Figures and Tables

**Table 1:** Sequences of the synthesized trichogin analogs. Modified residues are highlighted in bold.

*n*°	Peptide Sequence *^a^*
**trichogin**	*n*Oct-Aib-Gly^2^-Leu-Aib-Gly^5^-Gly-Leu-Aib^8^-Gly-Ile-Lol
**1**	*n*Oct-Aib-**Lys**^2^-Leu-Aib-**Lys**^5^-Gly-Leu-Aib^8^-Gly-Ile-Lol
**2**	*n*Oct-Aib-**Lys**^2^-Leu-Aib-Gly^5^-Gly-Leu-Aib^8^-Gly-Ile-Lol
**3**	*n*Oct-Aib-Gly^2^-Leu-Aib-**Lys**^5^-Gly-Leu-Aib^8^-Gly-Ile-Lol
**4**	*n*Oct-Aib-Gly^2^-Leu-Aib-**Lys**^5^-**Lys**-Leu-Aib^8^-Gly-Ile-Lol
**4c**	*n*Oct-Aib - **Lys**^2^-**Lys**-Leu-Aib^5^-Gly-Ile -Lol
**4r**	*n*Oct-Aib-Gly^2^-Leu-Aib-**Lys**^5^-**Lys**-Leu-Aib^8^-Gly-Ile-**Leu-NH_2_**
**4c1**	*n*Oct-Aib-Gly^2^-Leu-Aib-**Lys**^5^-**Lys** - **Leu-NH_2_**
**4c2**	*n*Oct-Aib - **Lys**^2^-**Lys**-Leu-Aib^5^-Gly-Ile-**Leu-NH_2_**
**5**	*n*Oct-Aib-Gly^2^-Leu-Aib-Gly^5^-**Lys**-Leu-Aib^8^-Gly-Ile-Lol
**6**	*n*Oct-Aib-Gly^2^-Leu-Aib-**Lys**^5^-**Aib**-Leu-Aib^8^-Gly-Ile-Lol
**7**	*n*Oct-Aib-**Lys**^2^-Leu-Aib-Gly^5^-**Lys**-Leu-Aib^8^-Gly-Ile-Lol
**8**	*n*Oct-Aib-Gly^2^-Leu-Aib-Gly^5^-Gly-Leu-**Api**^8^-Gly-Ile-Lol

*^a^ n*Oct, *n*-octanoyl; Aib, α-aminoisobutyric acid; Lol, leucinol; Api, 4-aminopiperidine-4-carboxylic acid.

**Table 2:** Inhibition of growth of *B. cinerea* in microtiter wells containing potato dextrose broth (PDB) medium after treatment with peptides at 15 µM.

Peptide	Growth Delay ^a^
trichogin	0 h
**1**	12–24 h
**2**	36–48 h
**3**	0 h
**4**	>7 days
**4r**	>7 days
**5**	>7 days
**6**	12–24 h
**7**	72–84 h
**8**	>7 days
Shorter analogs:	
**4c**	>7 days
**4c1**	>7 days
**4c2**	>7 days

^a^ Cultures were monitored for seven days in the presence of the resazurin vital dye. The color change of the dye indicated the start of growth. The growth delay is reported in comparison to the untreated control. The experiment was repeated three times obtaining similar results.

**Table 3:** Protection of grape berries from *B. cinerea* infection by treatment with **4** at 50 µM.

	DI ± SE ^a^	Asymptomatic Berries (%) ± SD ^a^
Control	1.93 ± 0.14	17.5 ± 8.7
Treated	1.03 * ± 0.24	51.25 * ± 18.0

^a^ Symptoms are expressed as Disease Index (DI) ± standard error (SE). The mean percentage of asymptomatic grape berries ± standard deviation (SD) was also calculated. Four independent infection experiments were performed for each treatment. Data were statistically analyzed by *t*-test. * indicates significant differences at *p* < 0.05.

**Table 4:** Comparison of the performances of Lys-containing analogs against *B. cinerea.*

Peptide	Lys Position(s)	Antifungal Activity ^a^
**1**	2 and 5	+ *^a^*
**2**	2	++
**3**	5	–
**4**	5 and 6	++++
**5**	6	++++
**6**	5 *(and Aib^6^)*	+
**7**	2 and 6	++

*^a^* +, levels of antifungal activity; –, no activity.

## Supplementary Materials

Supplementary Materials can be found at https://www.mdpi.com/1422-0067/21/20/7521/s1. Figures S1–S4, Structural models: Views both perpendicular and along the helix axis; Figures S5–S7, Antifungal activity and CD spectra; Peptide characterization: HPLC profiles, ESI-HRMS and NMR spectra.
