# Supplementary material for: Targeted Amino Acid Substitutions in a Trichoderma Peptaibol Confer Activity against Fungal Plant Pathogens and Protect Host Tissues from Botrytis cinerea Infection
Source: Int J Mol Sci. 2020 Oct 12;21(20):7521. doi: 10.3390/ijms21207521 (PMC7589190; doi:10.3390/ijms21207521)
Supplement: Supplementary file 1 [file ijms-21-07521-s001.pdf]

SUPPORTING INFORMATION

Targeted amino acid substitutions in a *Trichoderma* peptaibol confer activity against fungal plant pathogens and protect host tissues from *Botrytis cinerea* infection

M. De Zotti,*,[1] L. Sella,[2] A. Bolzonello,[2] L. Gabbatore,[1] C. Peggion,[1] A. Bortolotto,[1,2] I. Elmaghraby,[2,3] S. Tundo,[2] F. Favaron[2]

[1]*Department of Chemistry, University of Padova, Via Marzolo 1, 35131 Padova, Italy*

[2]*Department of Land, Environment, Agriculture and Forestry, University of Padova, Viale dell'Università 16, 35020, Legnaro (PD), Italy.*

[3]*Agricultural Research Center, Central Laboratory of Organic Agriculture, 9, Cairo Univ. St., Giza 12619, Egypt*

**E-mail: marta.dezotti@unipd.it*

Table of contents

1. Figures S1-S4 - Structural models: views both perpendicular and along the helix axis.

2. Figure S5-S7 - Antifungal activity and CD spectra

3. Peptide characterization: HPLC profiles, ESI-HRMS and NMR spectra.

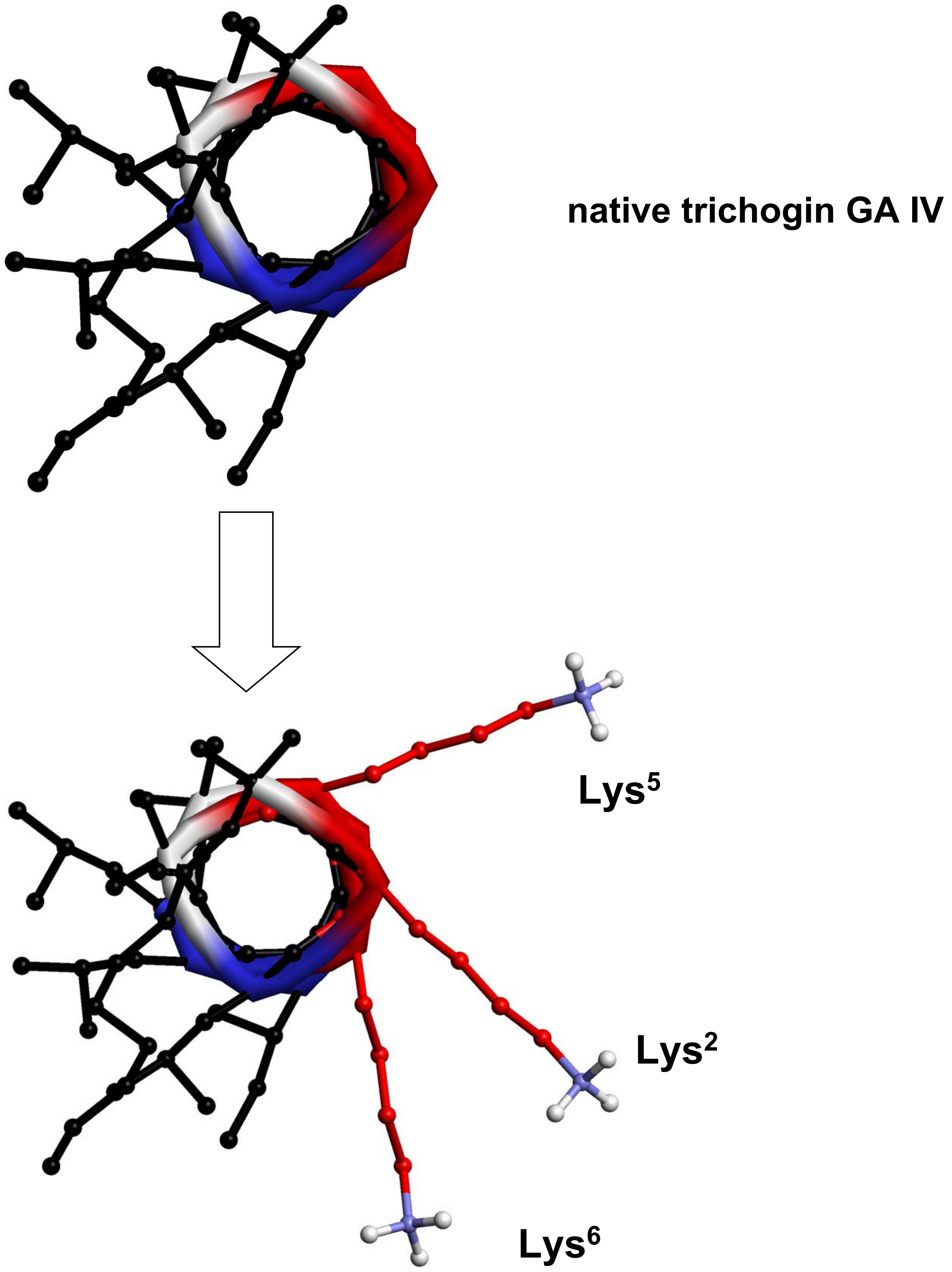

Figure S1. 3D-structure for compounds **1-5** and **7**. View along the helical axis.

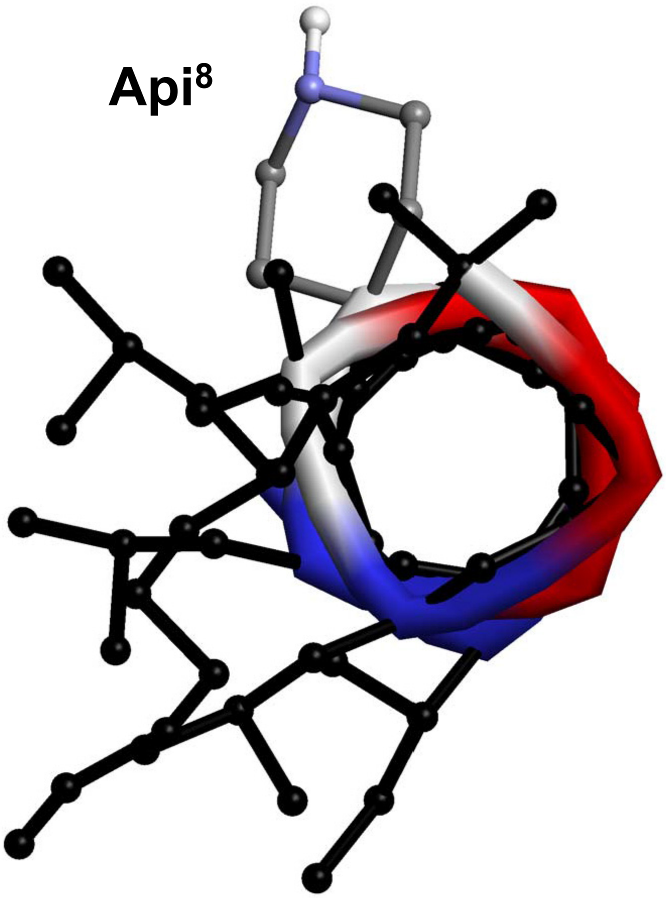

Figure S2. 3D-structure for compound **8**. View along the helical axis.

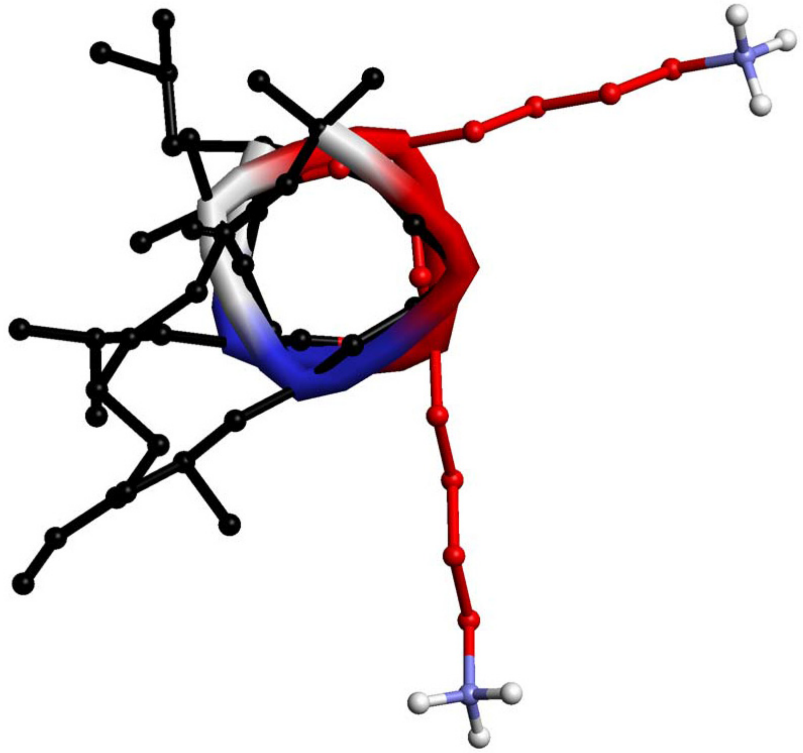

Figure S3. 3D-structure for compounds **4c** and **4c1**. View along the helical axis.

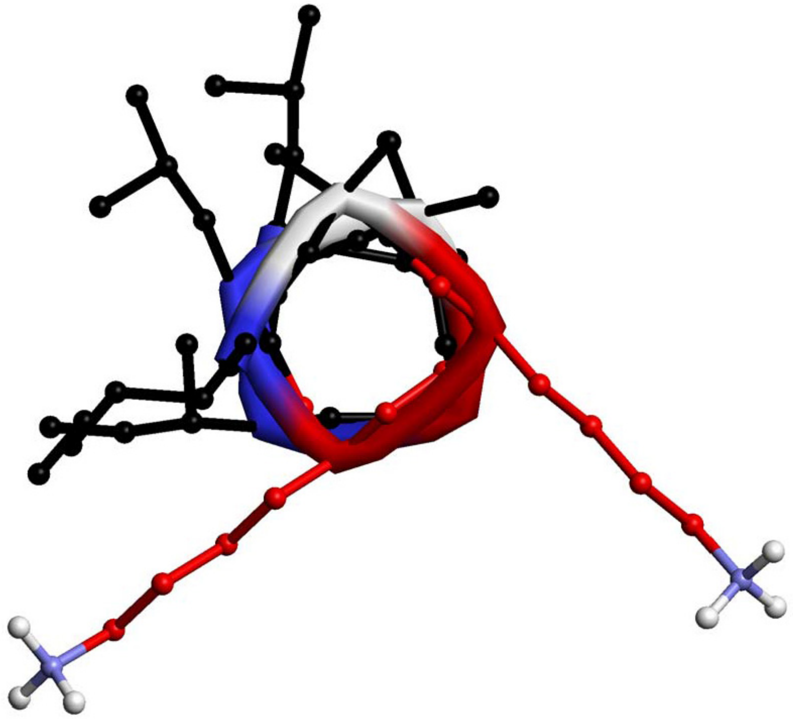

Figure S4. 3D-structure for compound **4c2**. View along the helical axis.

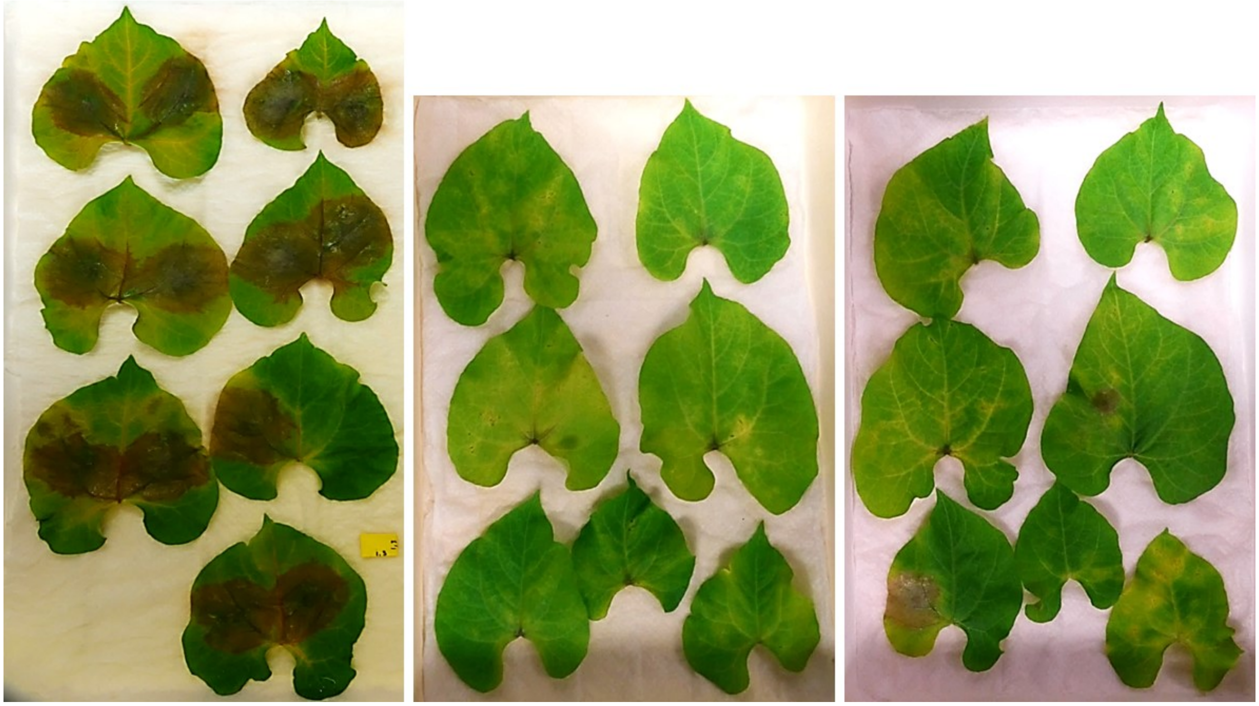

Untreated control **50 µM peptide 4** **15 µM peptide 4**

Fig. S5. Representative symptoms produced by *Botrytis cinerea* on common bean leaves (Borlotto var. Teggia) untreated or treated with the peptide **4** at 50 µM or 15 µM at 7 days post inoculation (dpi). Leaves were arranged on moist towels in plastic trays and inoculated on two or three points with 10 µL of a conidial suspension containing 2.5 x 10^6 mL^{-1} *B. cinerea* conidia in potato dextrose broth (PDB).

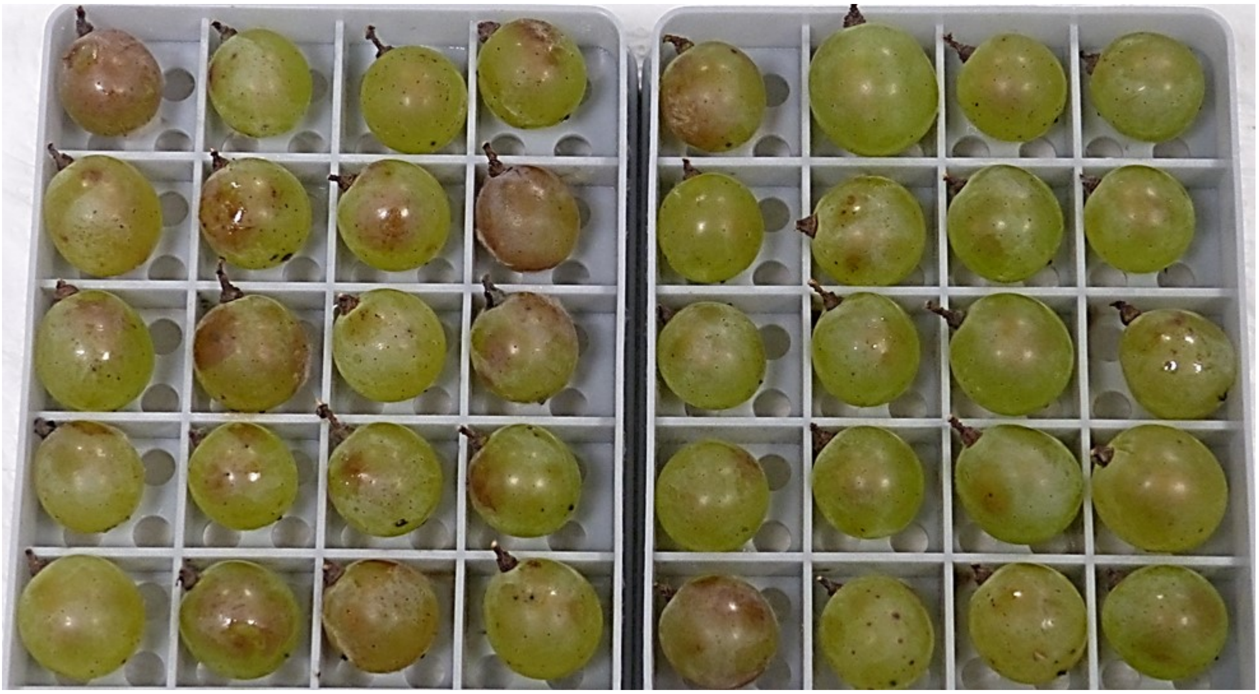

Untreated control **50 µM peptide 4**

Fig. S6. Representative symptoms produced by *Botrytis cinerea* on grape berries (cv. Glera) untreated or treated with the peptide **4** at 50 µM at 4 days post inoculation (dpi). Ripe grape berries were surface-sterilized, rinsed with sterile water, separately arranged on a plastic tray and sprayed with *B. cinerea* conidia at a concentration of 1.85 x 10^6 mL^{-1}.

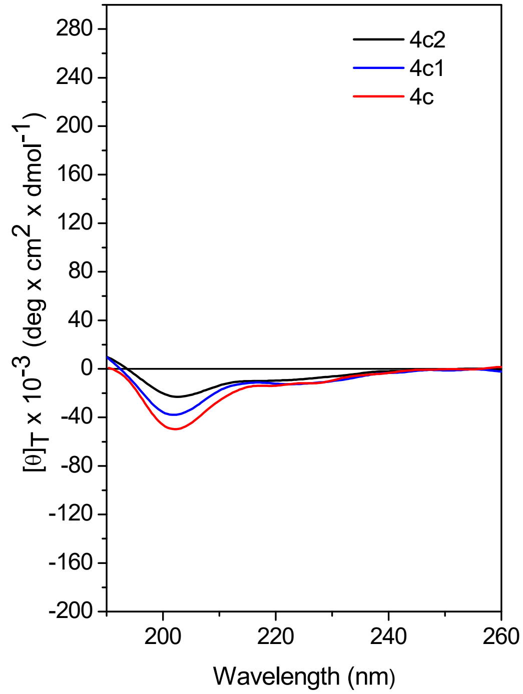

Fig. S7. CD profiles acquired for peptides **4c**, **4c1** and **4c2** in aqueous solution (peptide concentration: 10^{-4} M).

Peptide Characterization (HPLC, ESI-HRMS, ^{1}H NMR)

Table S1. Sequences of the synthesized trichogin analogs.

n°	peptide sequence[a]
**1**	*n*Oct-Aib-**Lys**^{2}-Leu-Aib-**Lys**^{5}-Gly-Leu-Aib-Gly-Ile-Lol
**2**	*n*Oct-Aib-**Lys**^{2}-Leu-Aib-Gly^{5}-Gly-Leu-Aib-Gly-Ile-Lol
**3**	*n*Oct-Aib-Gly^{2}-Leu-Aib-**Lys**^{5}-Gly-Leu-Aib-Gly-Ile-Lol
**4**	*n*Oct-Aib-Gly^{2}-Leu-Aib-**Lys-Lys**-Leu-Aib-Gly-Ile- Lol
**4c**	*n*Oct-Aib - **Lys**^{2}-**Lys**-Leu-Aib-Gly-Ile- Lol
**4r**	*n*Oct-Aib-Gly^{2}-Leu-Aib-**Lys**^{5}-**Lys**-Leu-Aib-Gly-Ile-**Leu-NH$_2$**
**4c1**	*n*Oct-Aib-Gly^{2}-Leu-Aib-**Lys**^{5}-**Lys**-Leu - **Leu-NH$_2$**
**4c2**	*n*Oct-Aib - **Lys**^{2}-**Lys**-Leu-Aib-Gly-Ile- **Leu-NH$_2$**
**5**	*n*Oct-Aib-Gly^{2}-Leu-Aib-Gly^{5}-**Lys**-Leu-Aib-Gly-Ile- Lol
**6**	*n*Oct-Aib-Gly^{2}-Leu-Aib-**Lys**^{5}-**Aib**-Leu-Aib-Gly-Ile- Lol
**7**	*n*Oct-Aib-**Lys**^{2}-Leu-Aib-Gly^{5}-**Lys**-Leu-Aib-Gly-Ile- Lol
**8**	*n*Oct-Aib-Gly^{2}-Leu-Aib-Gly^{5}-Gly-Leu-**Api^{8}**-Gly-Ile-Lol

[a] *n*Oct, *n*-octanoyl; Aib, α-aminoisobutyric acid; Lol, leucinol; Api, 4-aminopiperidine-4-carboxylic acid.

Abbreviations:

ESI, Electron Spray Ionization

HRMS, High Resolution Mass Spectrometry

Eluants for HPLC:

A: H_2O/CH_3CN 9:1 + 0.05% TFA

B: CH_3CN/H_2O 9:1 + 0.05% TFA

peptide **1** (**K25**)

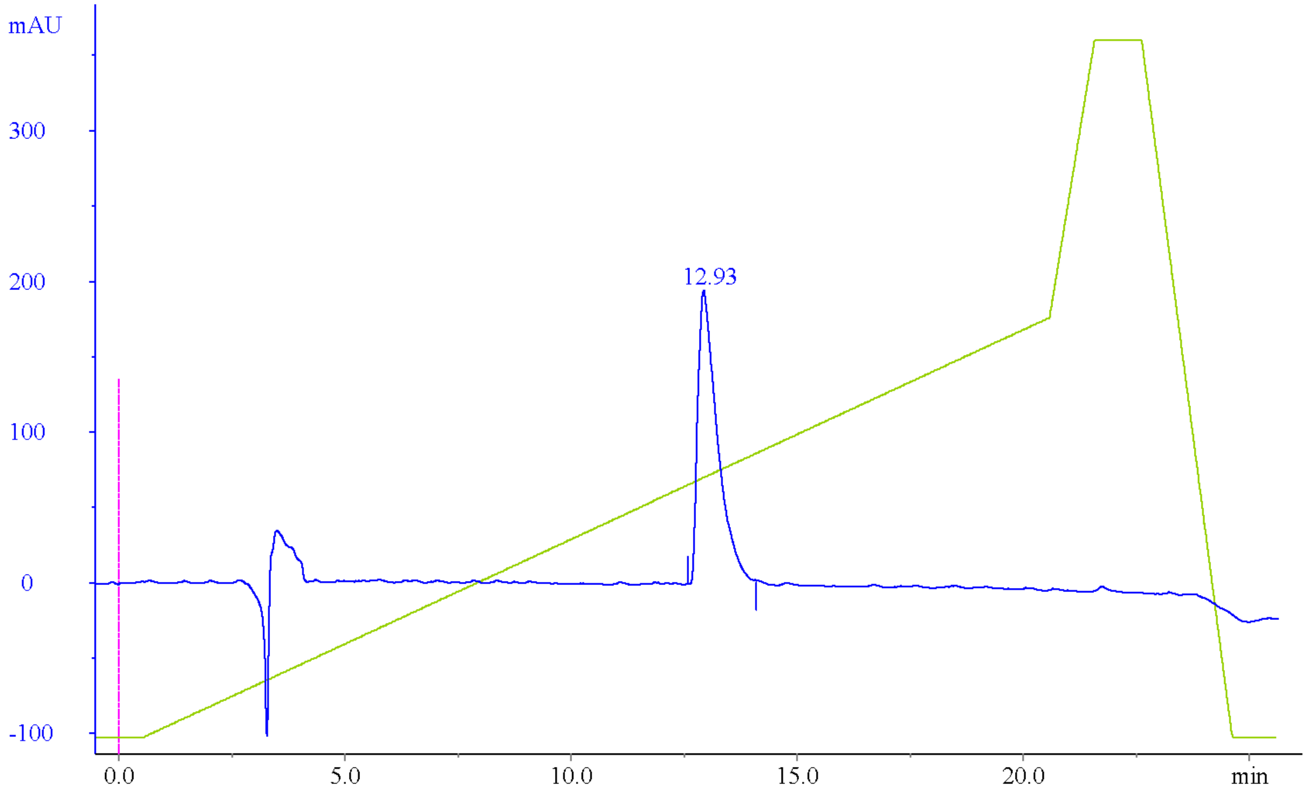

HPLC profile obtained for peptide **1** (gradient: 50→80%B [CH_3CN/H_2O 9:1 + 0.05% TFA] in 20 minutes; flow rate: 1mL/min; C_{18} Phenomenex 300Å) Retention time (R_t) = 12.93 min. Detection Wavelength: 216 nm.

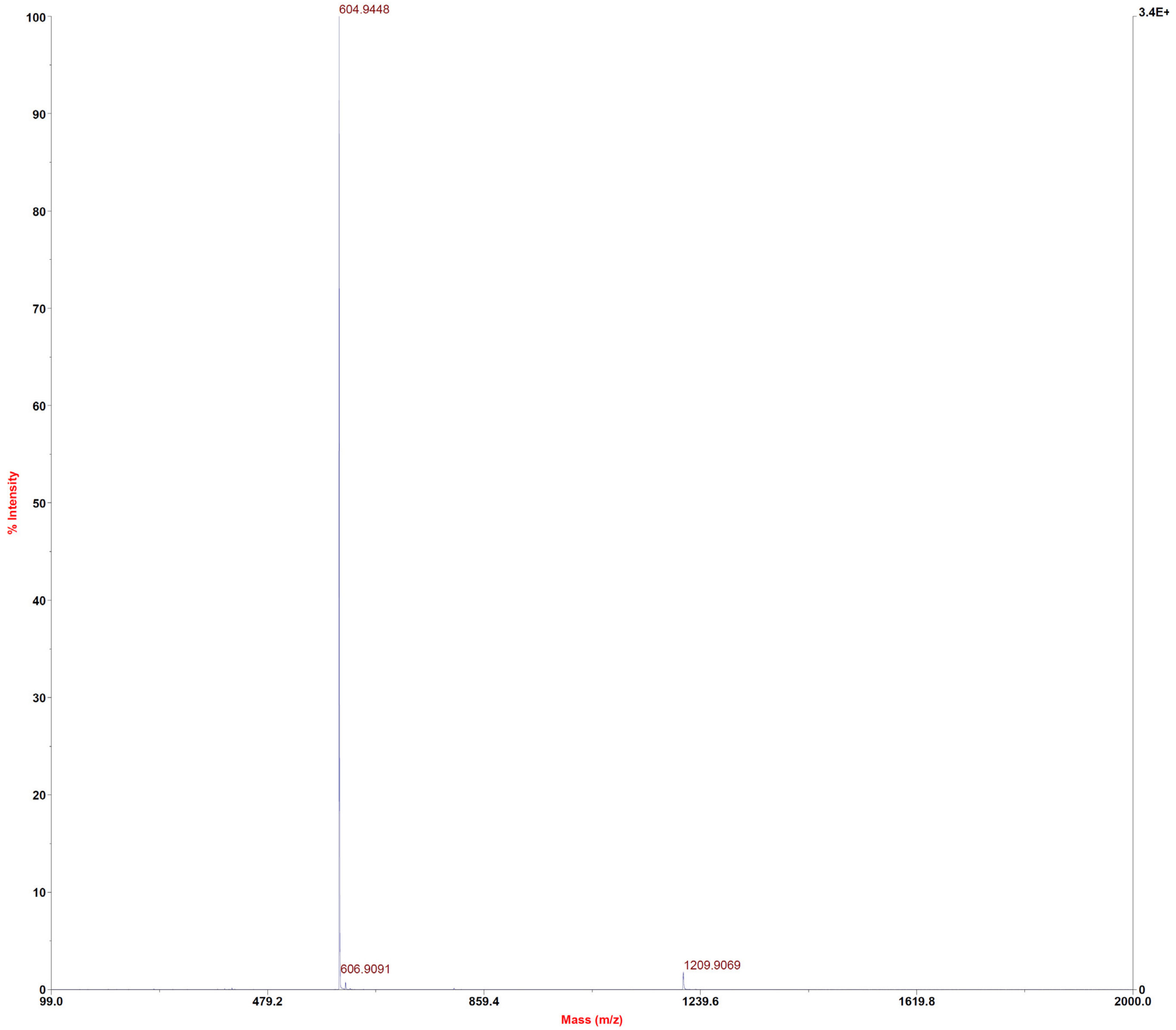

ESI-HRMS for peptide **1**: $[M+H]^{+}_{calcd.}$ 1208.8632; $[M+H]^{+}_{found}$: 1208.8896 (*monoisotopic*), $[M+2H]^{2+}_{found}$: 604.9448.

peptide **2** (**K2**)

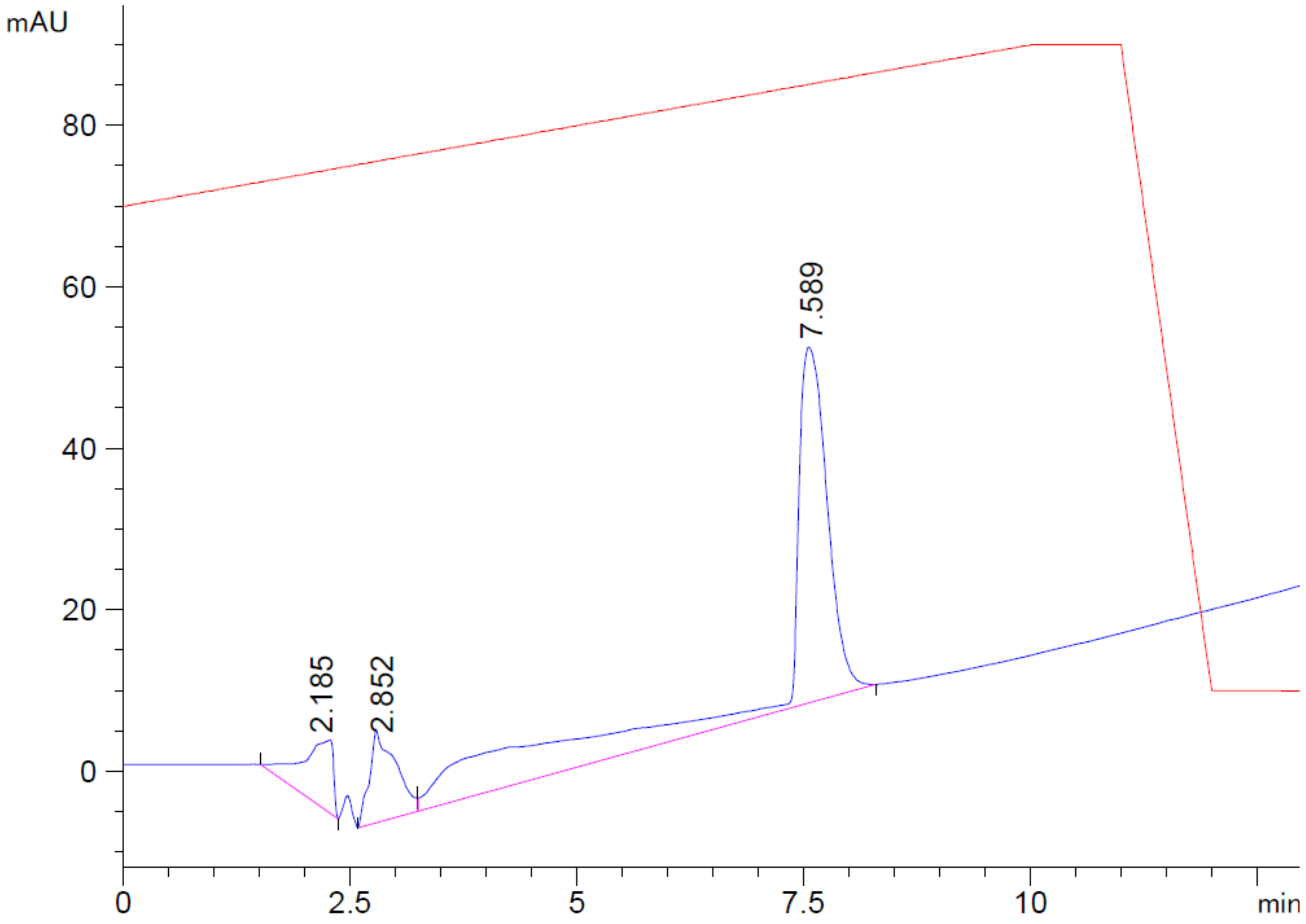

HPLC profile obtained for peptide **2** (gradient: 70→90%B [B: CH_3CN/H_2O 9:1 + 0.05% TFA] in 10 minutes; flow rate: 1mL/min; C_{18} Phenomenex 100Å) Retention time (R_t) = 7.59 min. Detection Wavelength: 226 nm.

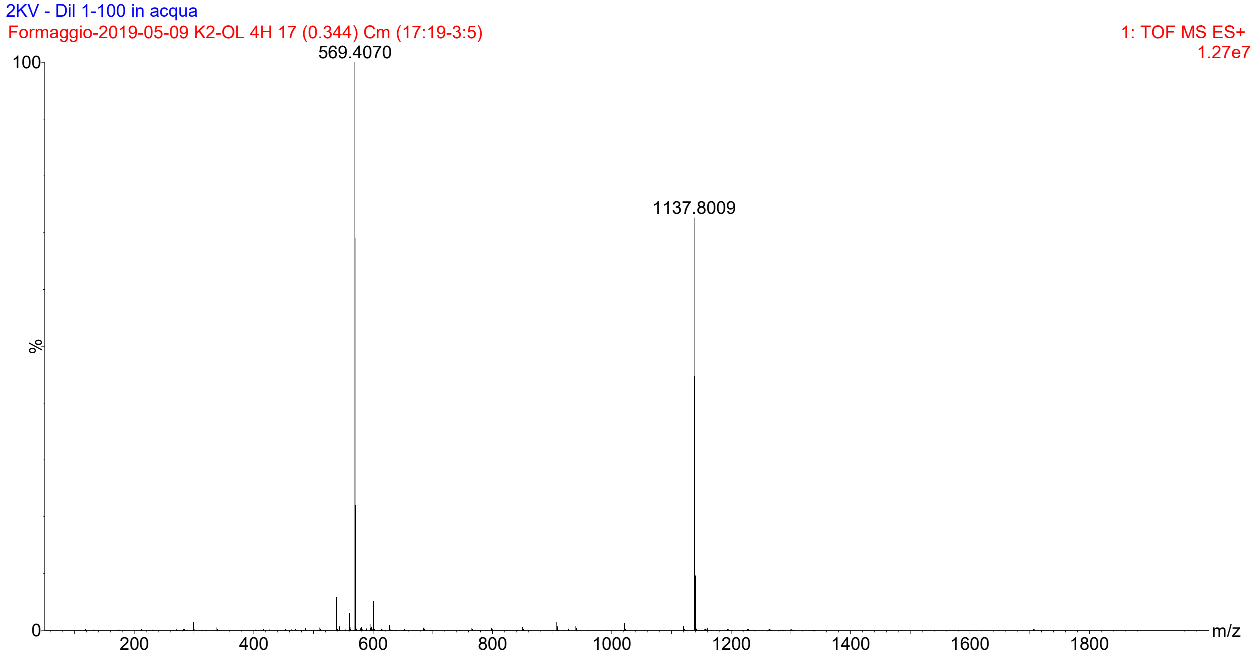

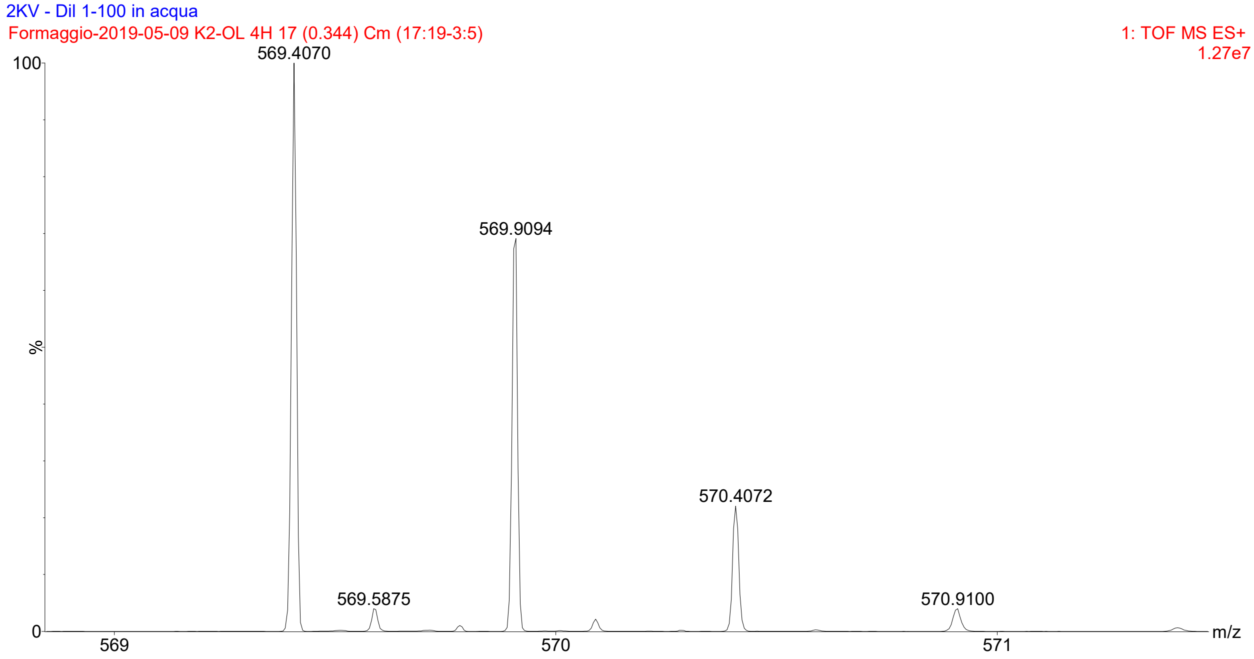

ESI-HRMS for peptide **2**: $[M+H]^{+}_{calcd.}$ 1137.7997; $[M+H]^{+}_{found}$: 1137.8009 (*monoisotopic*), $[M+2H]^{2+}_{found}$: 569.4070.

peptide **3** (**K5**)

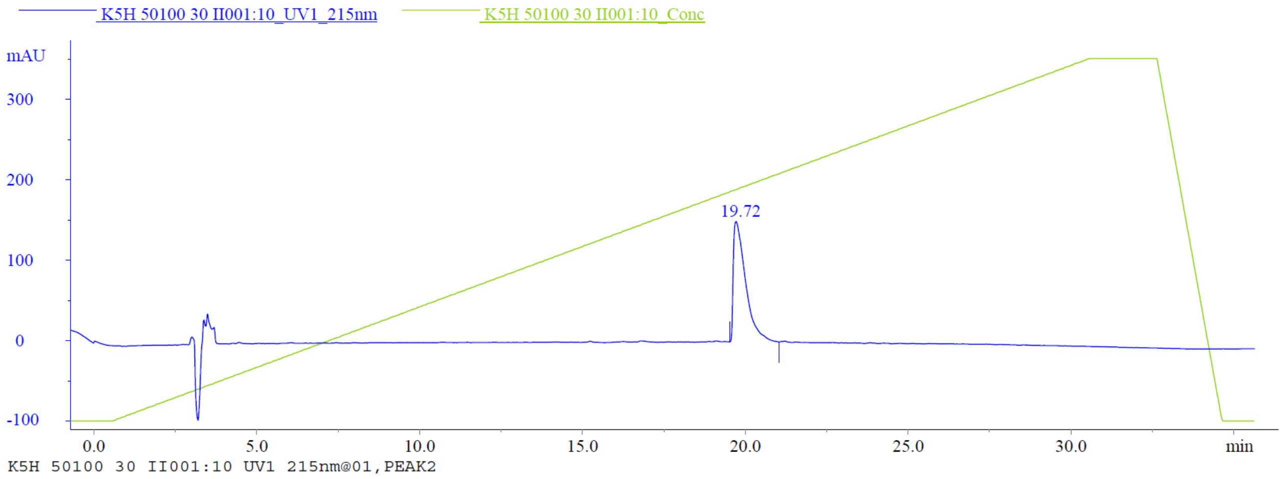

HPLC profile obtained for peptide **3** (gradient: 50→100%B [B: CH_3CN/H_2O 9:1 + 0.05% TFA] in 30 minutes; flow rate: 1mL/min; C_{18} Phenomenex 300Å) Retention time (R_t) = 19.72 min. Detection Wavelength: 215 nm.

Applied Biosystems Mariner System 5220

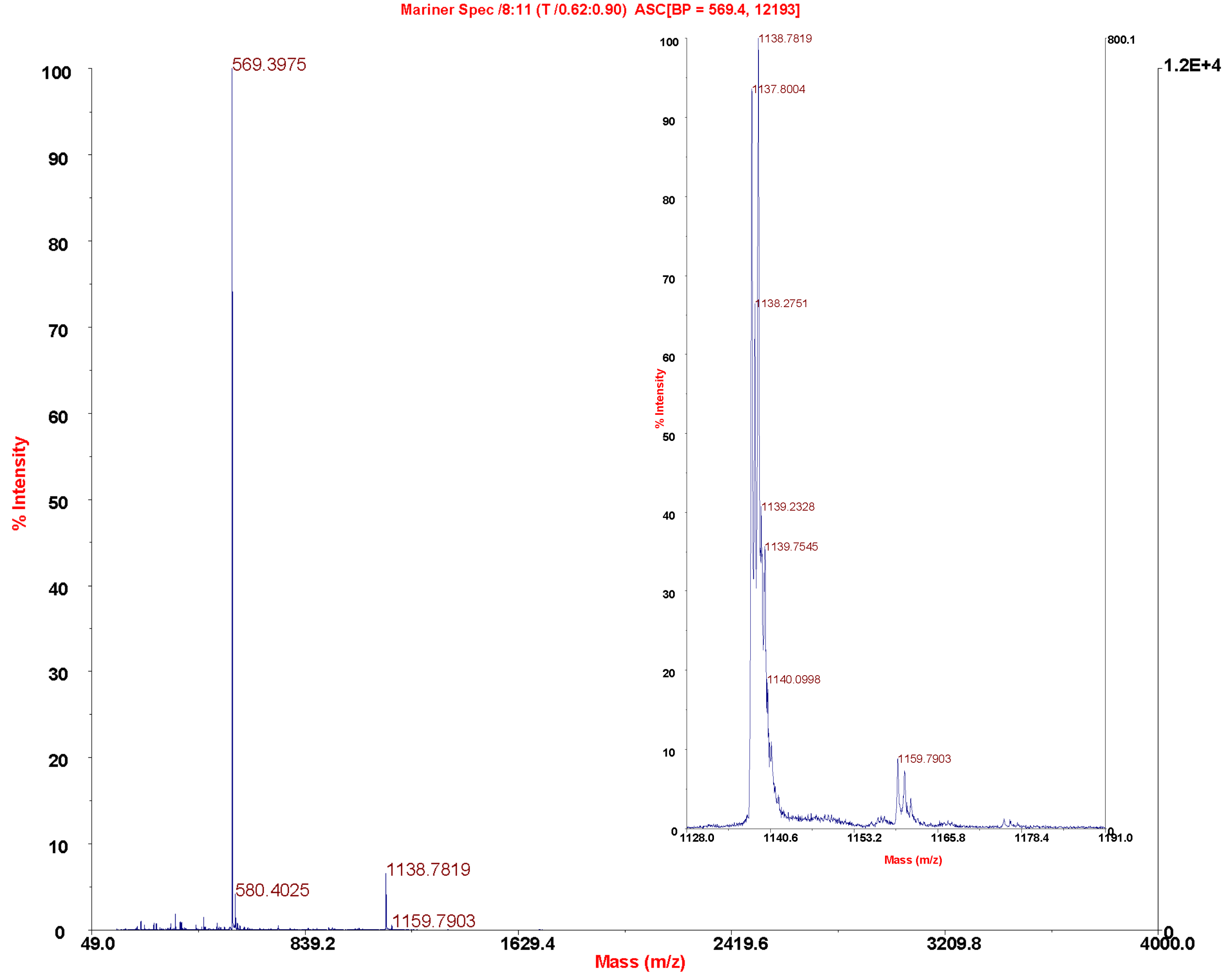

ESI-HRMS for peptide **3**: $[M+H]^+_{calcd.}$ 1137.7997; $[M+H]^+_{found}$: 1137.8004 (*monoisotopic*), $[M+Na]^+_{found}$: 1159.7903 (*monoisotopic*), $[M+2H]^{2+}_{found}$: 569.3975.

peptide **4** (**K56**)

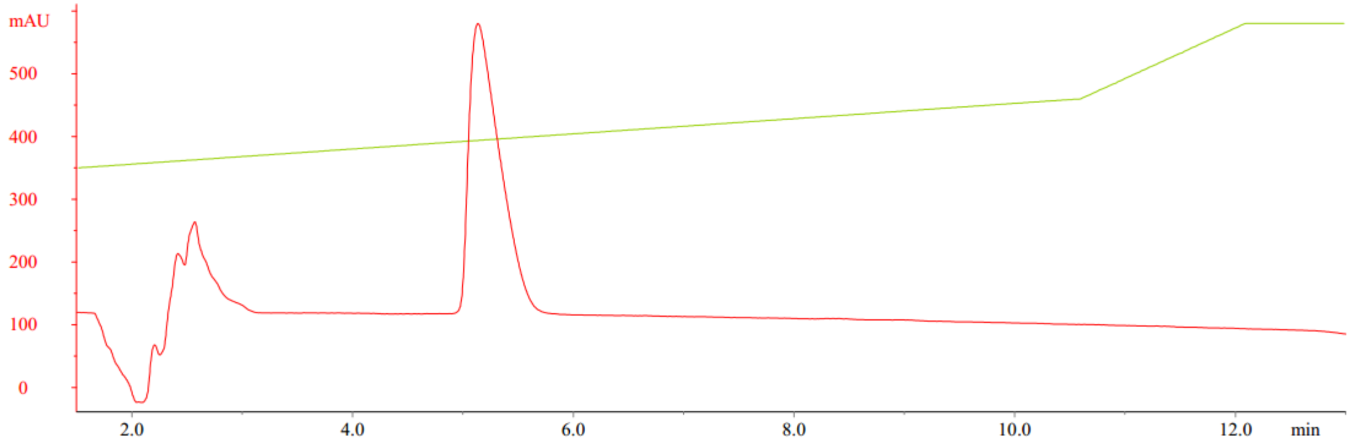

HPLC profile obtained for peptide **4** (gradient: 60→80%B [B: CH_3CN/H_2O 9:1 + 0.05% TFA] in 10 minutes; flow rate: 1mL/min; C_{18} Agilent Zorbax RX-C18) Retention time (R_t) = 5.14 min. Detection Wavelength: 226 nm.

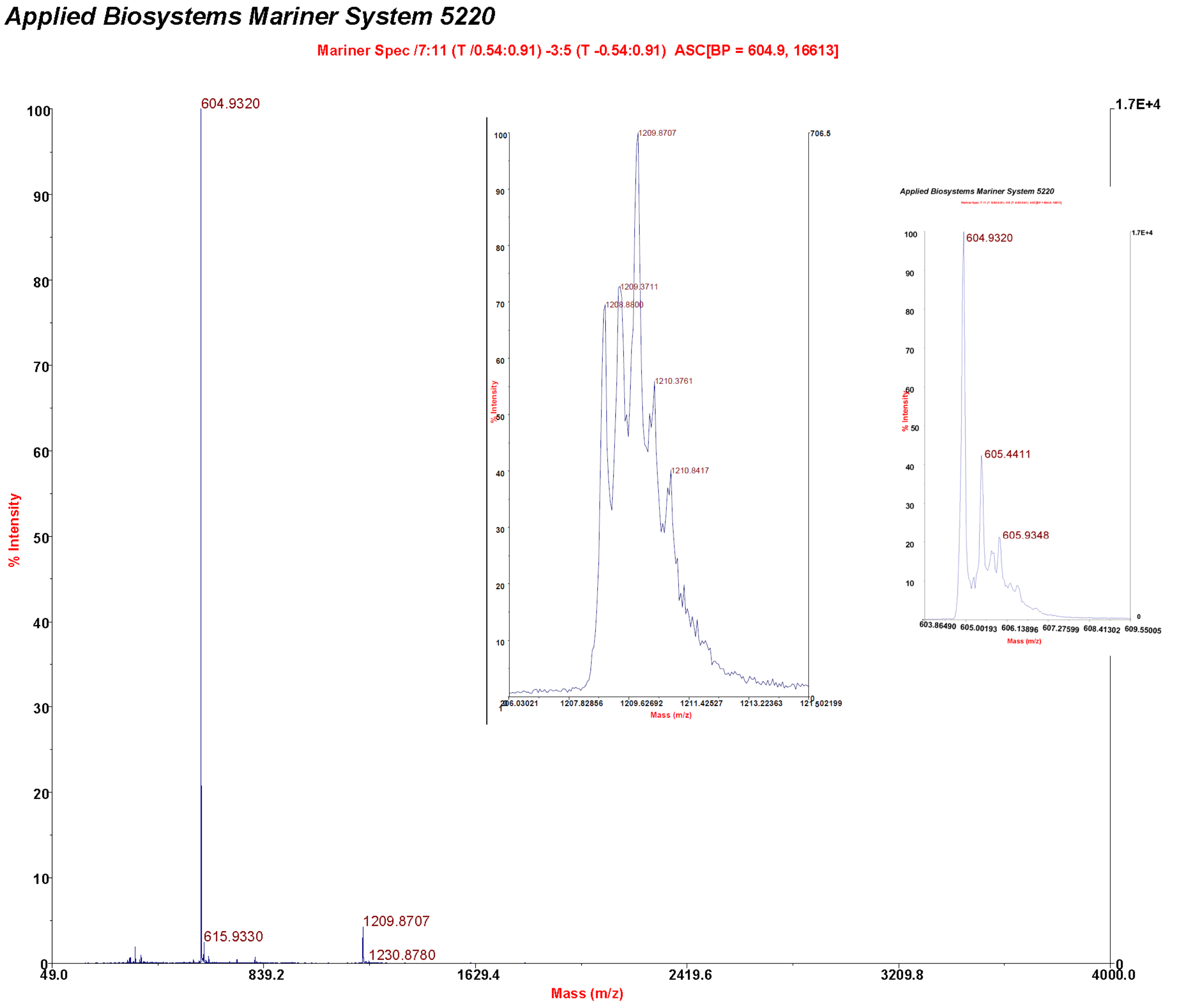

ESI-HRMS for peptide **4**: $[M+H]^+_{calcd.}$ 1208.8732; $[M+H]^+_{found}$: 1208.8800 (*monoisotopic*), $[M+Na]^+_{found}$: 1230.8780 (*monoisotopic*), $[M+2H]^{2+}_{found}$: 604.9320.

peptide **4r** (**K56-NH2**)

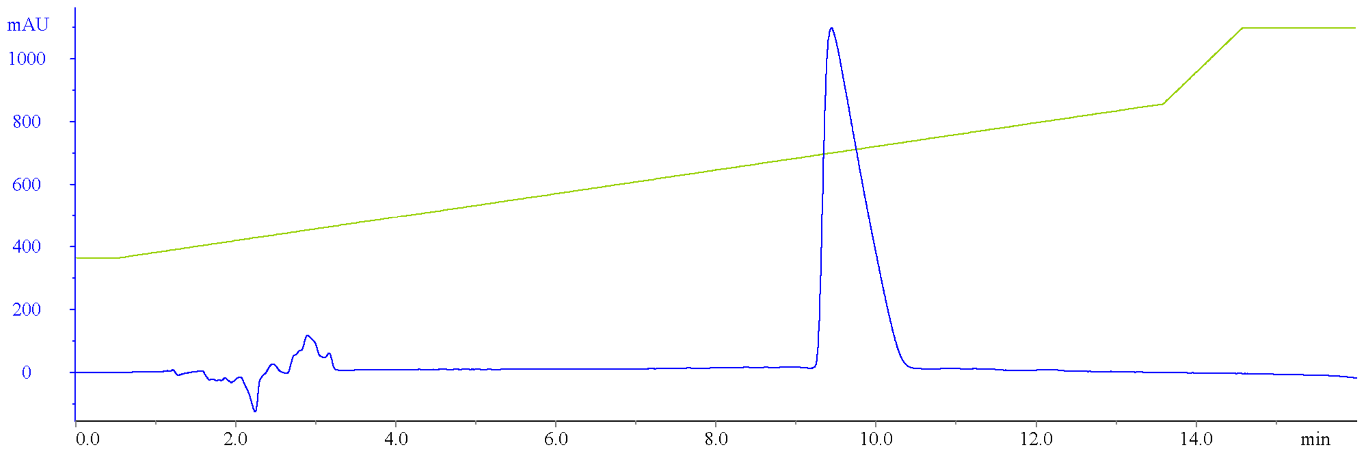

HPLC profile obtained for peptide **4r** (gradient: 40→80%B [CH_3CN/H_2O 9:1 + 0.05% TFA] in 15 minutes; flow rate: 1mL/min; C_{18} Phenomenex 300Å) Retention time (R_t) = 9.44 min. Detection Wavelength: 208 nm.

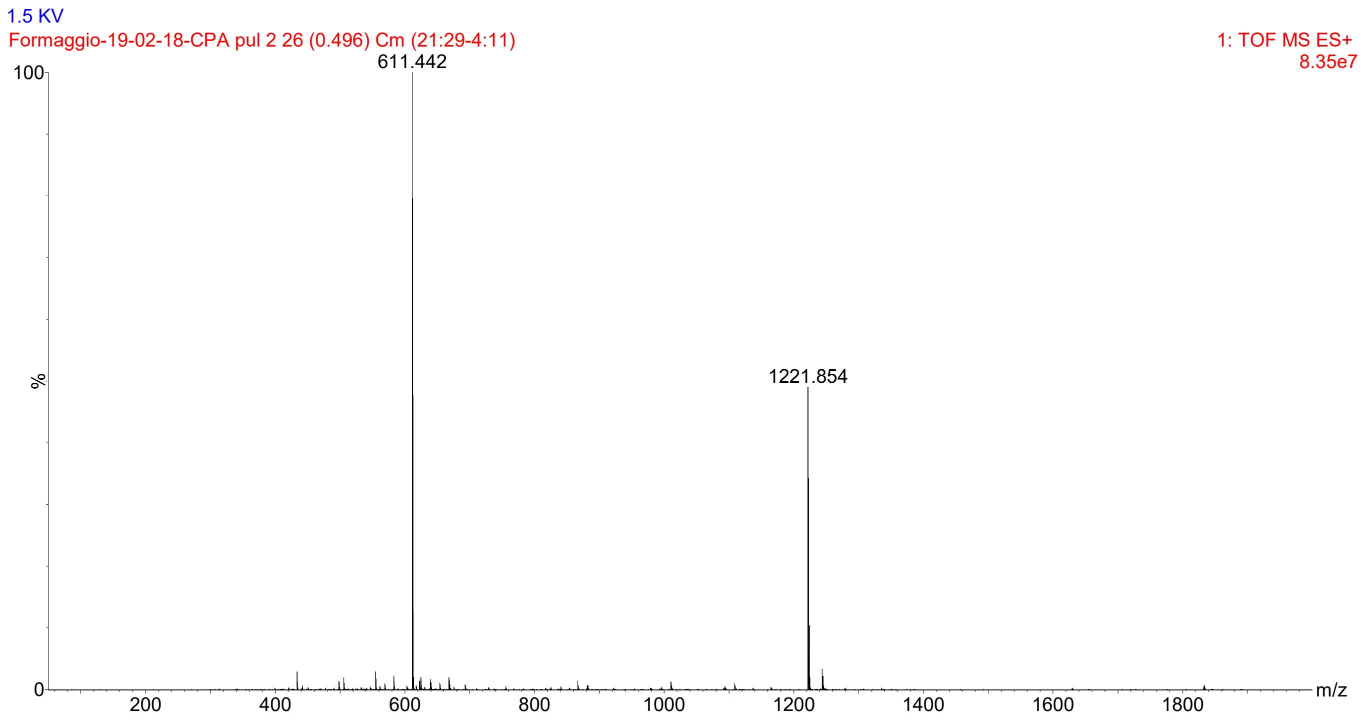

ESI-HRMS for peptide **4r**: $[M+H]^+_{calcd.}$ 1221.8584; $[M+H]^+_{found}$: 1221.8540 (*monoisotopic*), $[M+2H]^{2+}_{found}$: 611.4420.

peptide **4c**

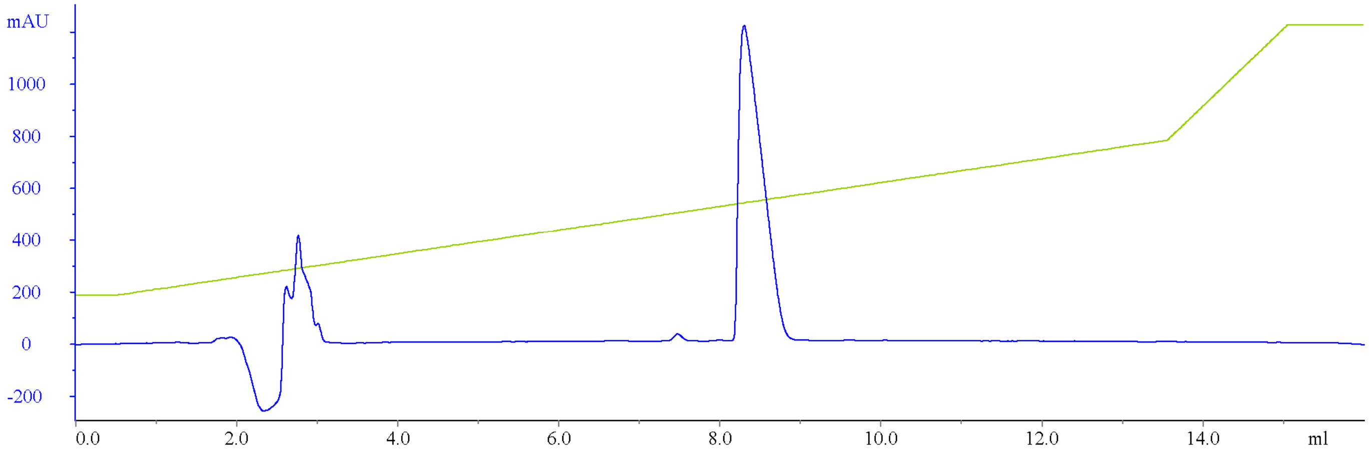

HPLC profile obtained for peptide **4c** (gradient: 30→70%B [CH_3CN/H_2O 9:1 + 0.05% TFA] in 15 minutes; flow rate: 1mL/min; C_{18} Phenomenex 300Å) Retention time (R_t) = 8.31 min. Detection Wavelength: 208 nm.

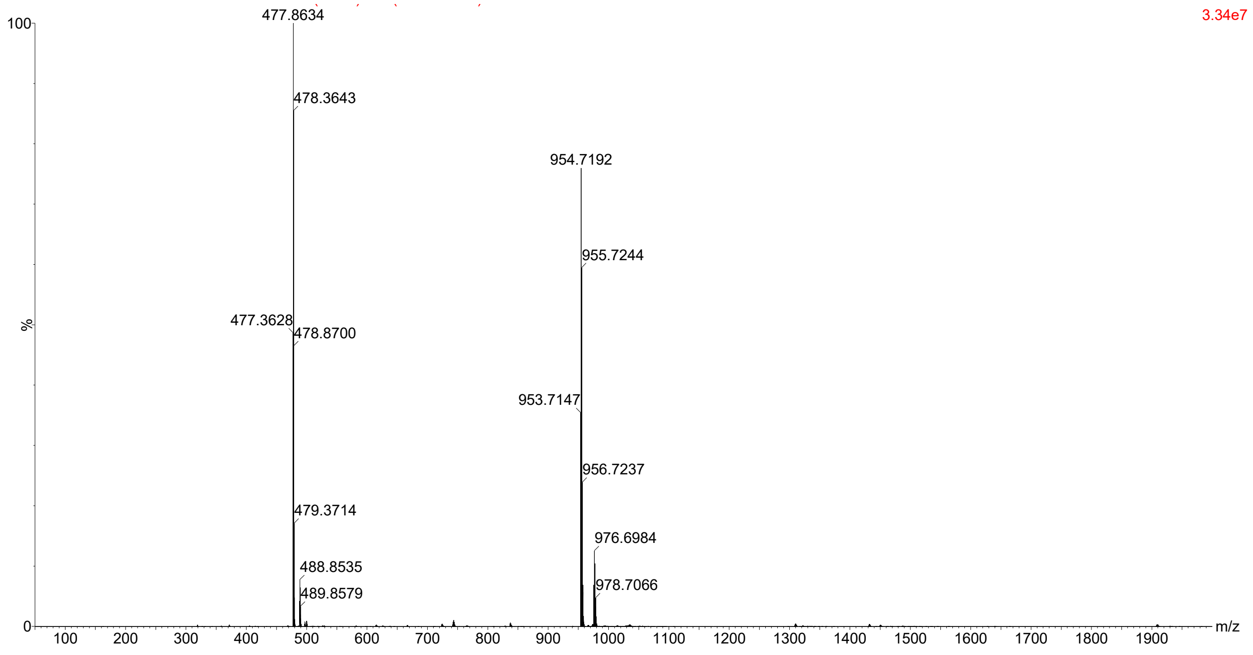

ESI-MS for peptide **4c**: $[M+H]^+_{calcd.}$ 953.7049; $[M+H]^+_{found}$: 953.7147 (*monoisotopic*) $[M+Na]^+_{found}$: 975.6951, $[M+2H^{2+}]_{found}$: 477.3628.

peptide **4c1** (**Oct-UGLUKKL-NH$_2$**)

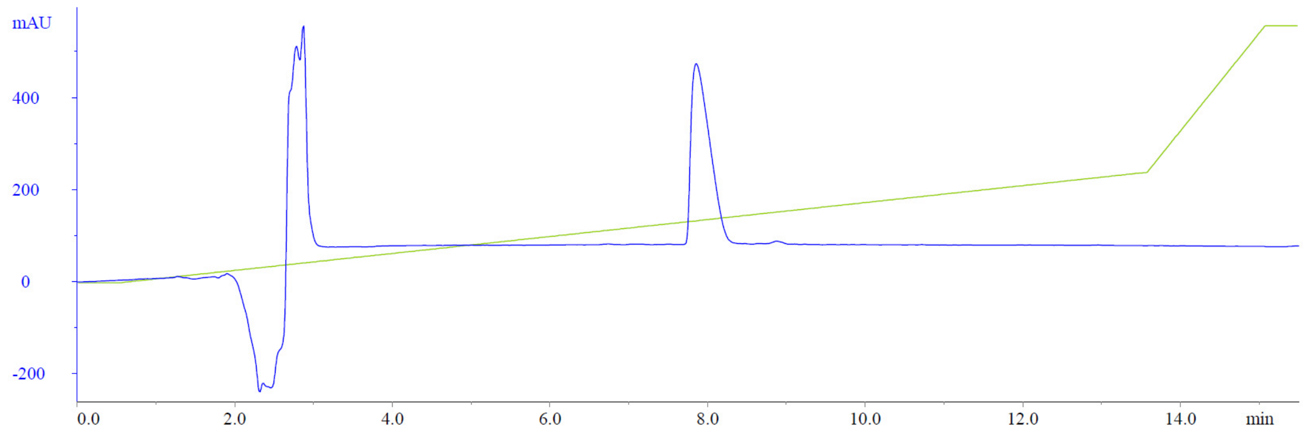

HPLC profile obtained for peptide **4c1** (gradient: 30→60%B [CH_3CN/H_2O 9:1 + 0.05% TFA] in 14 minutes; flow rate: 1 mL/min; C_{18} Phenomenex 300Å) Retention time (R_t) = 7.85 min. Detection Wavelength: 208 nm.

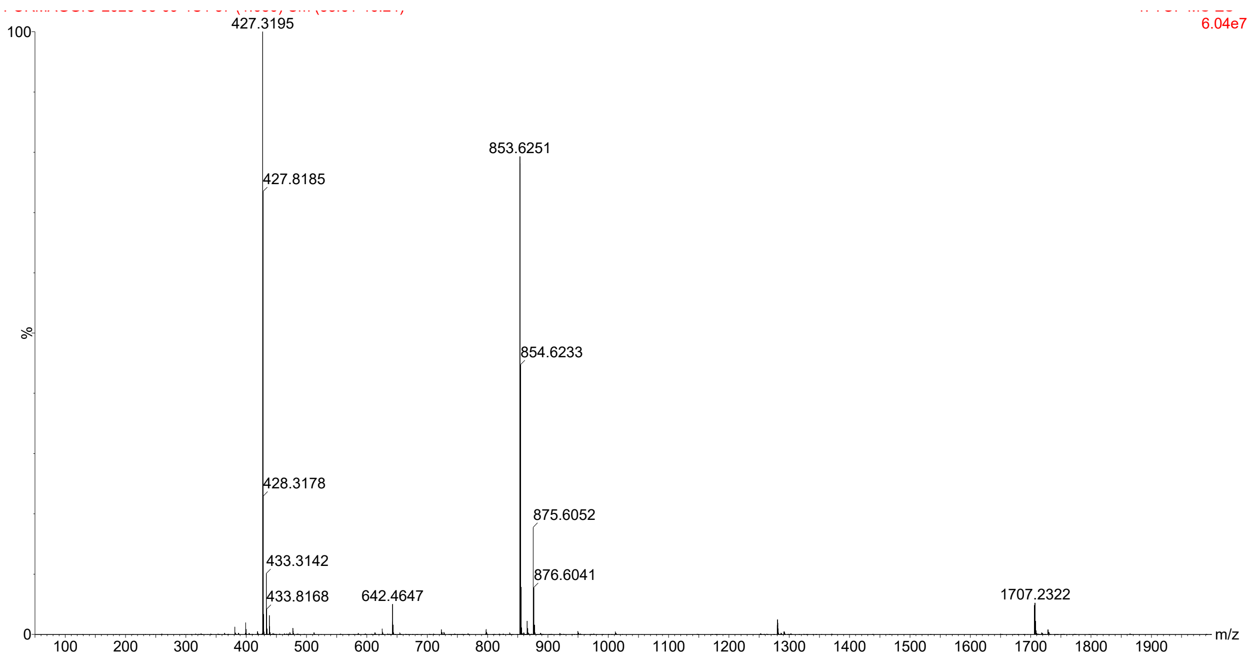

ESI-MS for peptide **4c1**: $[M+H]^+_{calcd.}$: 853.6261; $[M+H]^+_{found}$: 853.6251, $[M+2H^{2+}]_{found}$: 427.3195. $[2M+H]^+_{found}$: 1707.2322.

peptide **4c2** (**Oct-AibLLAibGIL-NH_2**)

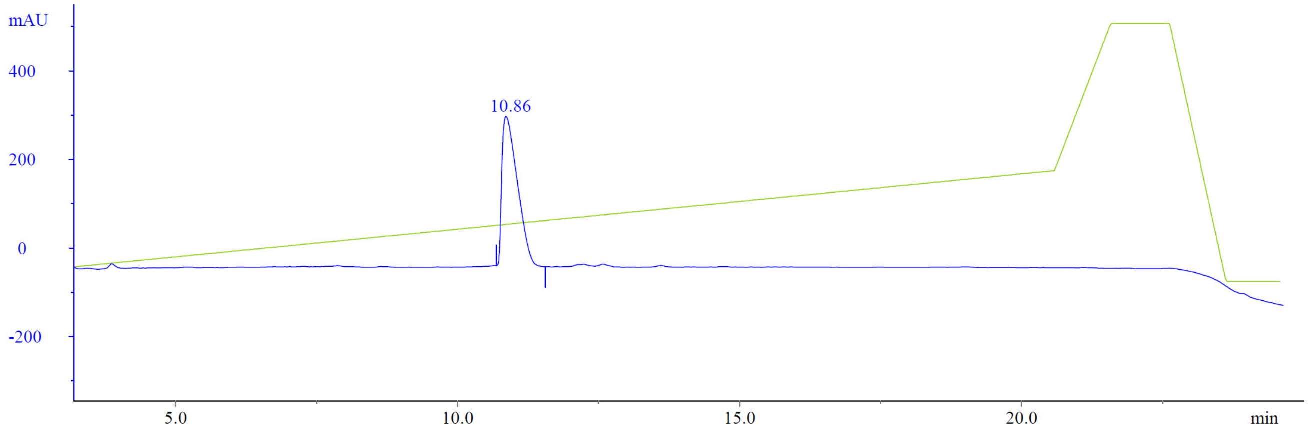

HPLC profile obtained for peptide **4c2** (gradient: 30→60%B [CH_3CN/H_2O 9:1 + 0.05% TFA] in 20 minutes; flow rate: 1mL/min; C_{18} Phenomenex 300Å) Retention time (R_t) = 10.86 min. Detection Wavelength: 208 nm.

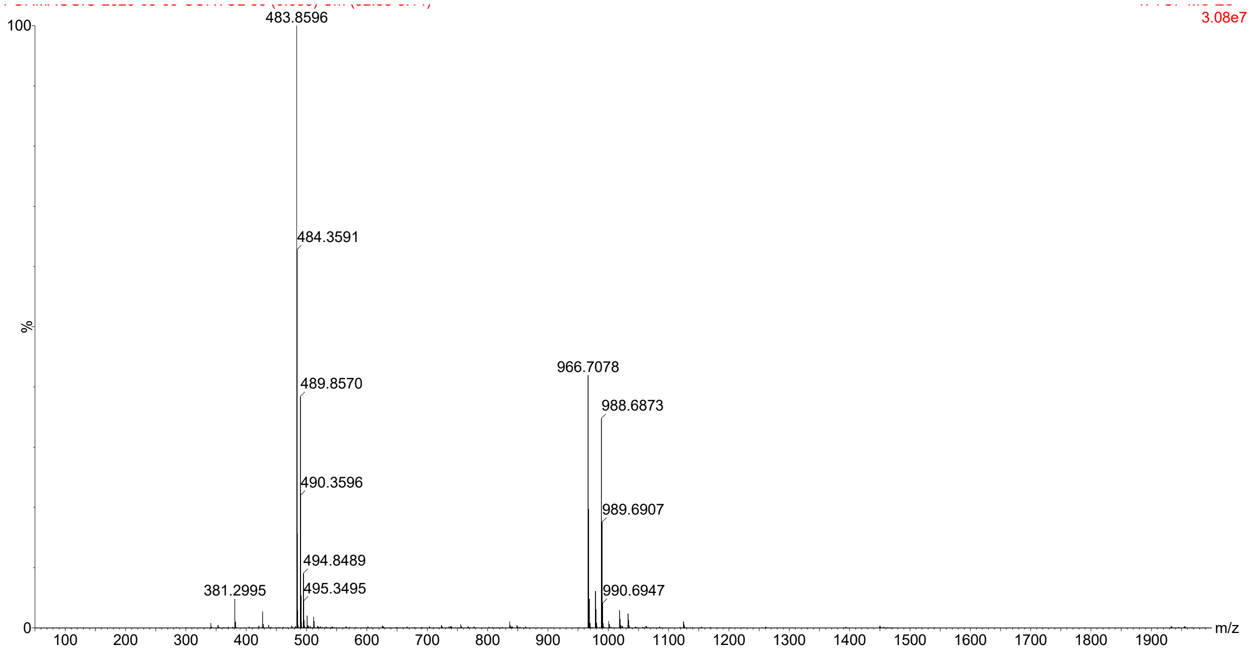

ESI-MS for peptide **4c2**: $[M+H]^+_{calcd.}$ 966.7001; $[M+H]^+_{found}$: 966.7078, $[M+Na]^+_{found}$: 988.6873, $[M+2H^{2+}]_{found}$: 483.8596.

peptide **5** (**K6**)

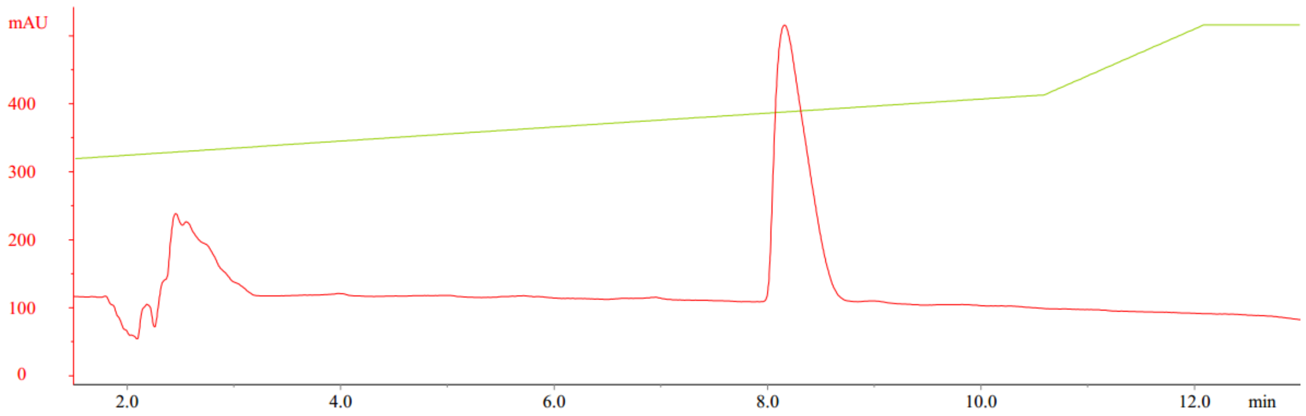

HPLC profile obtained for peptide **5** (gradient: 60→80%B [CH_3CN/H_2O 9:1 + 0.05% TFA] in 10 minutes; flow rate: 1mL/min; C_{18} Agilent Zorbax RX-C18) R_t = 8.16 min. Detection Wavelength: 216 nm.

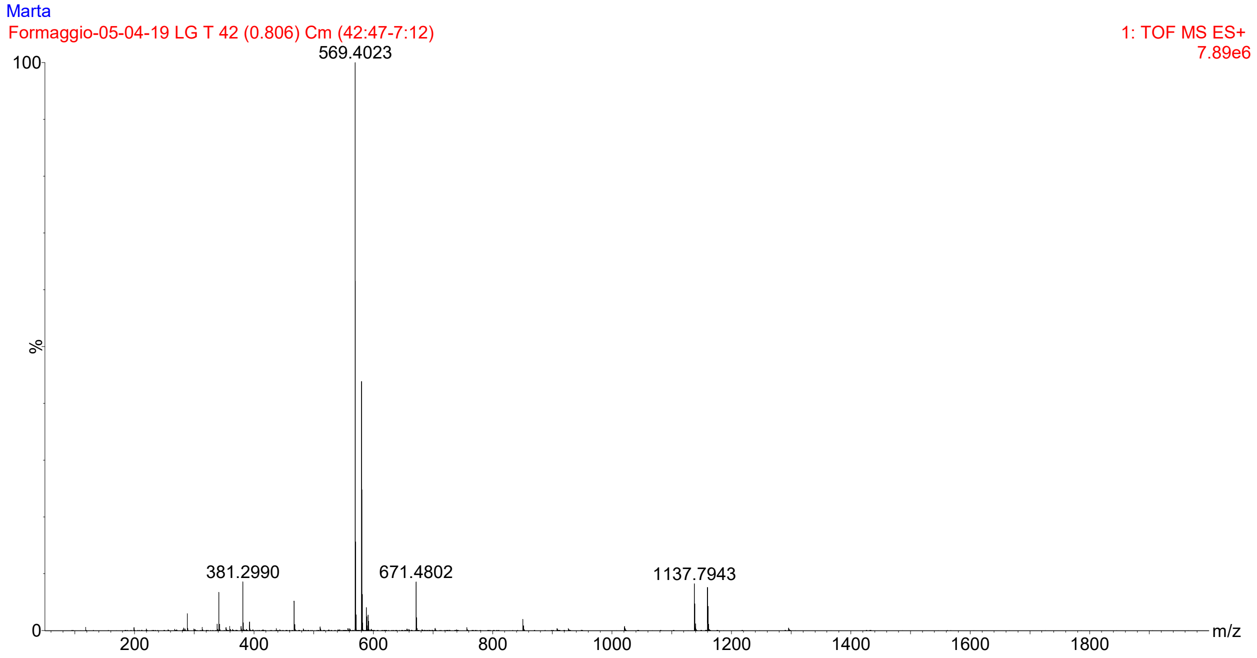

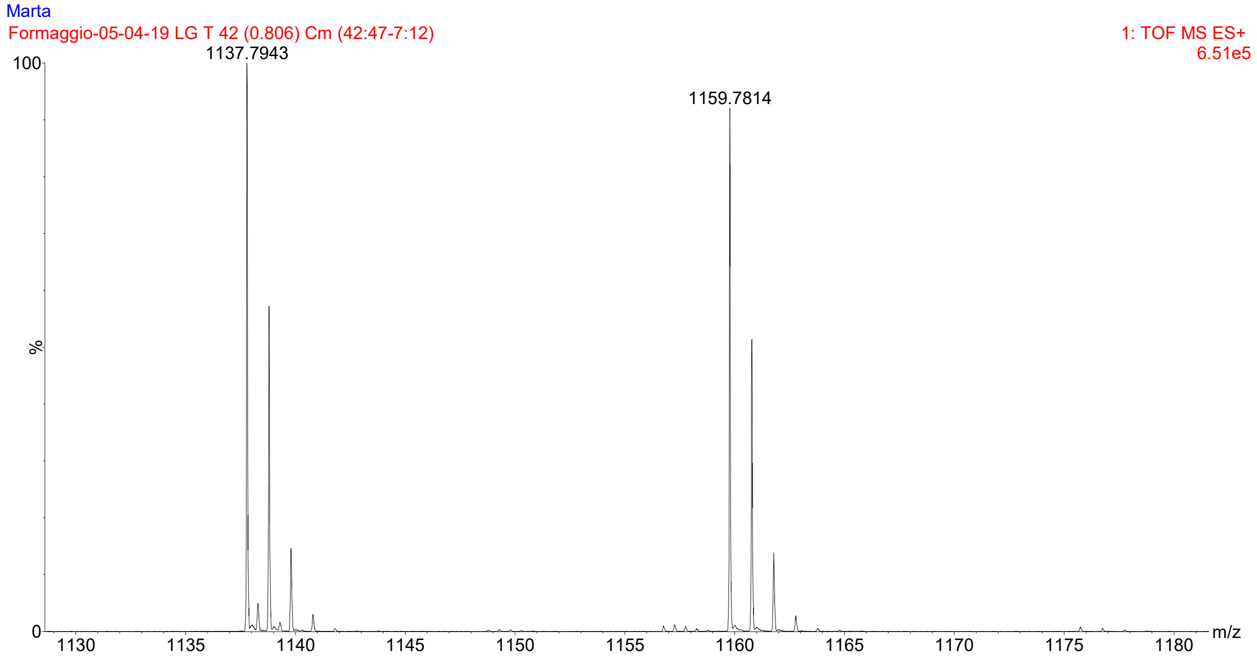

ESI-HRMS for peptide **5**: $[M+H]^{+}_{calcd.}$ 1137.7997; $[M+H]^{+}_{found}$: 1137.7943 (*monoisotopic*), $[M+Na]^{+}_{found}$: 1159.7814 (*monoisotopic*), $[M+2H]^{2+}_{found}$:569.4023. *Peaks at 381.2990 and 671.4802 are impurities from samples injected before (the instrument was contaminated).*

peptide **6** (**K5U6**)

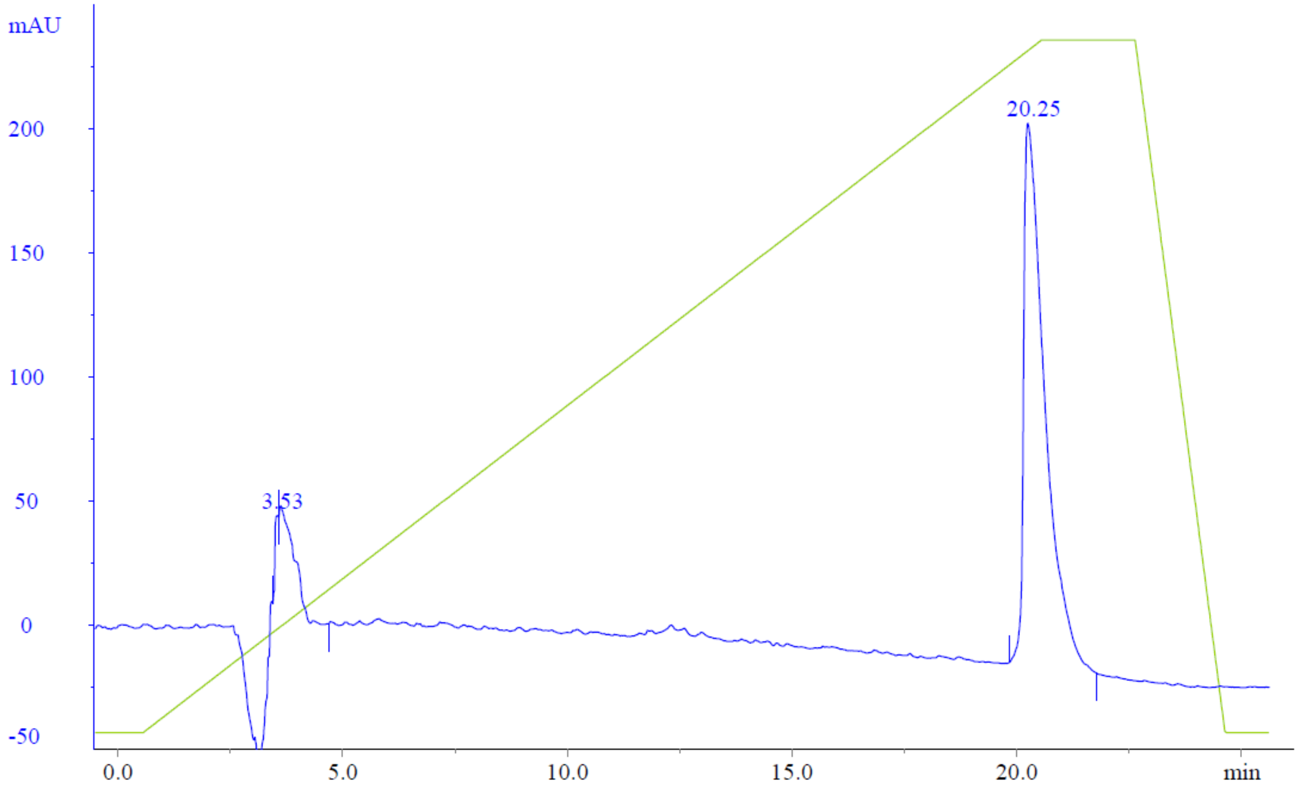

HPLC profile obtained for peptide **6** (gradient: 60→100%B [CH_3CN/H_2O 9:1 + 0.05% TFA] in 20 minutes; flow rate: 1mL/min; C_{18} Phenomenex 300Å) R_t = 20.25 min. Detection Wavelength: 216 nm.

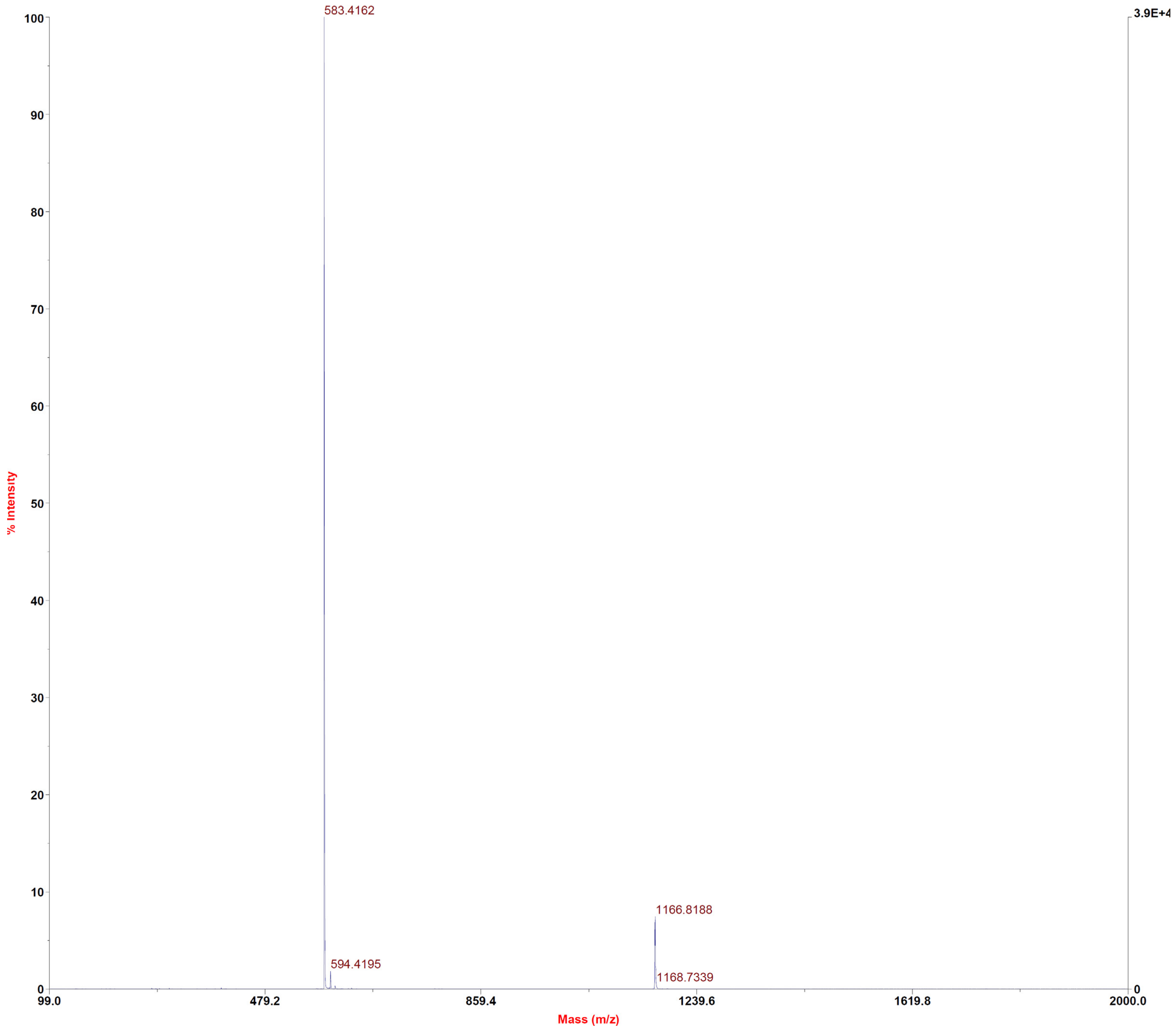

ESI-MS for peptide **6**: $[M+H]_{calcd.}$ 1165.8210; $[M+H]_{found}$: 1165.8271 (*monoisotopic*), $[M+2H^{2+}]_{found}$:583.4162, $[M+H+Na^{2+}]_{found}$: 594.4195.

peptide **7** (**K26**)

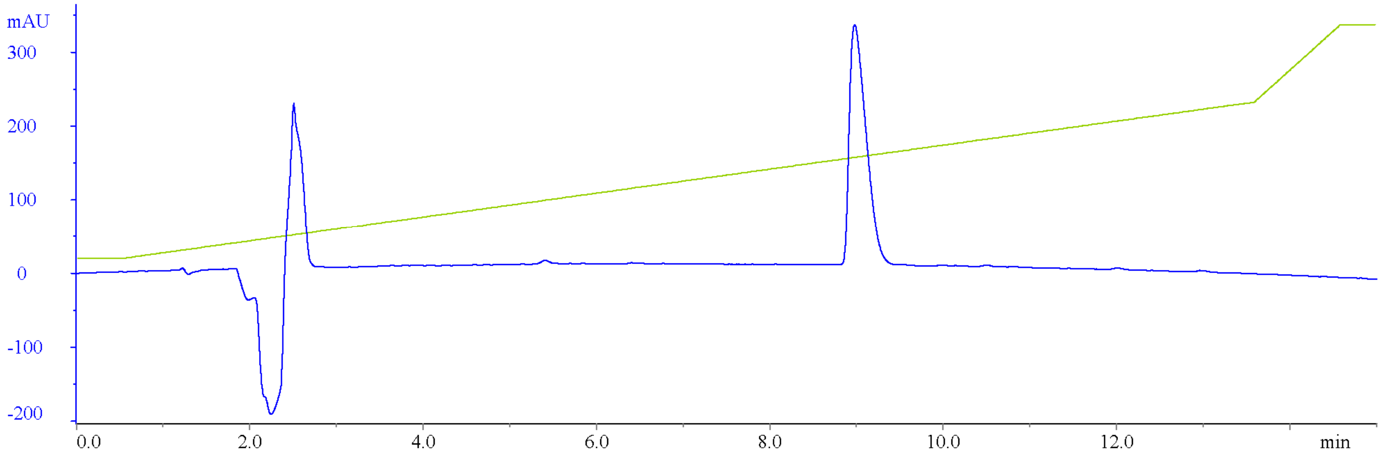

HPLC profile obtained for peptide **7** (gradient: 40→80%B [CH_3CN/H_2O 9:1 + 0.05% TFA] in 15 minutes; flow rate: 1mL/min; C_{18} Phenomenex 300Å) R_t = 8.97 min. Detection Wavelength: 208 nm.

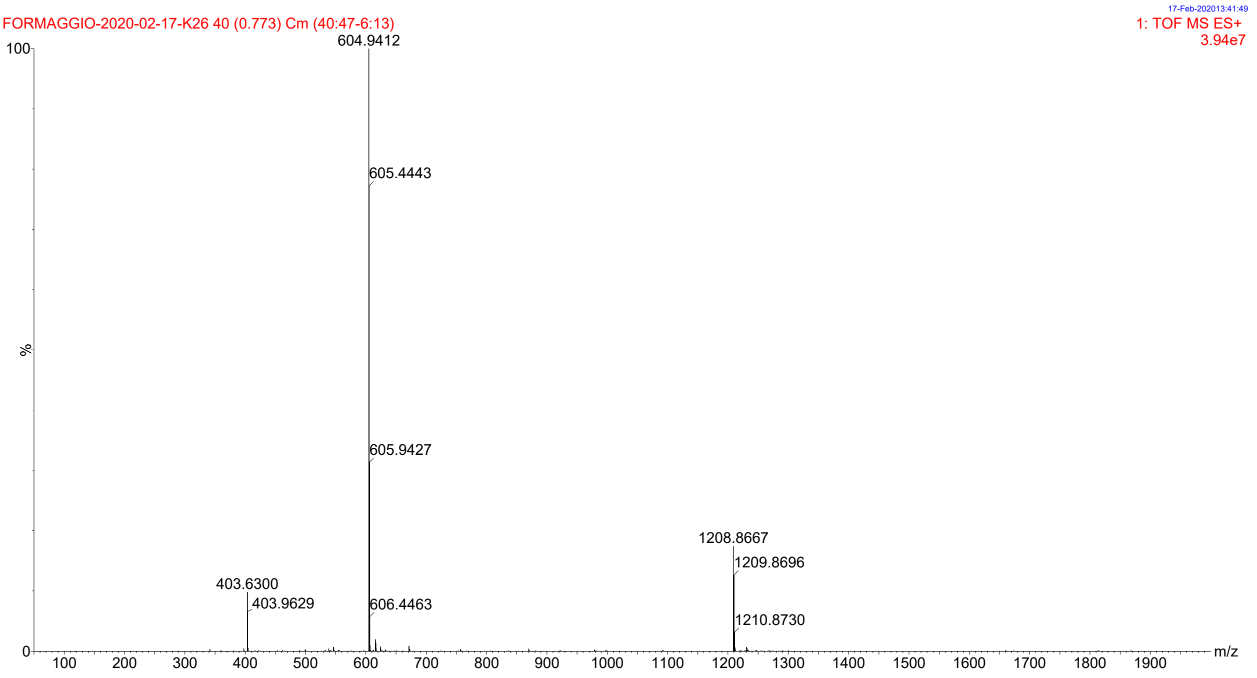

ESI-MS for peptide **7**: $[M+H]_{calcd.}$ 1208.8632; $[M+H]_{found}$: 1208.8667, $[M+2H^{2+}]_{found}$:604.9412; $[M+3H^{3+}]_{found}$: 403.6300.

peptide **8** (**Api8**)

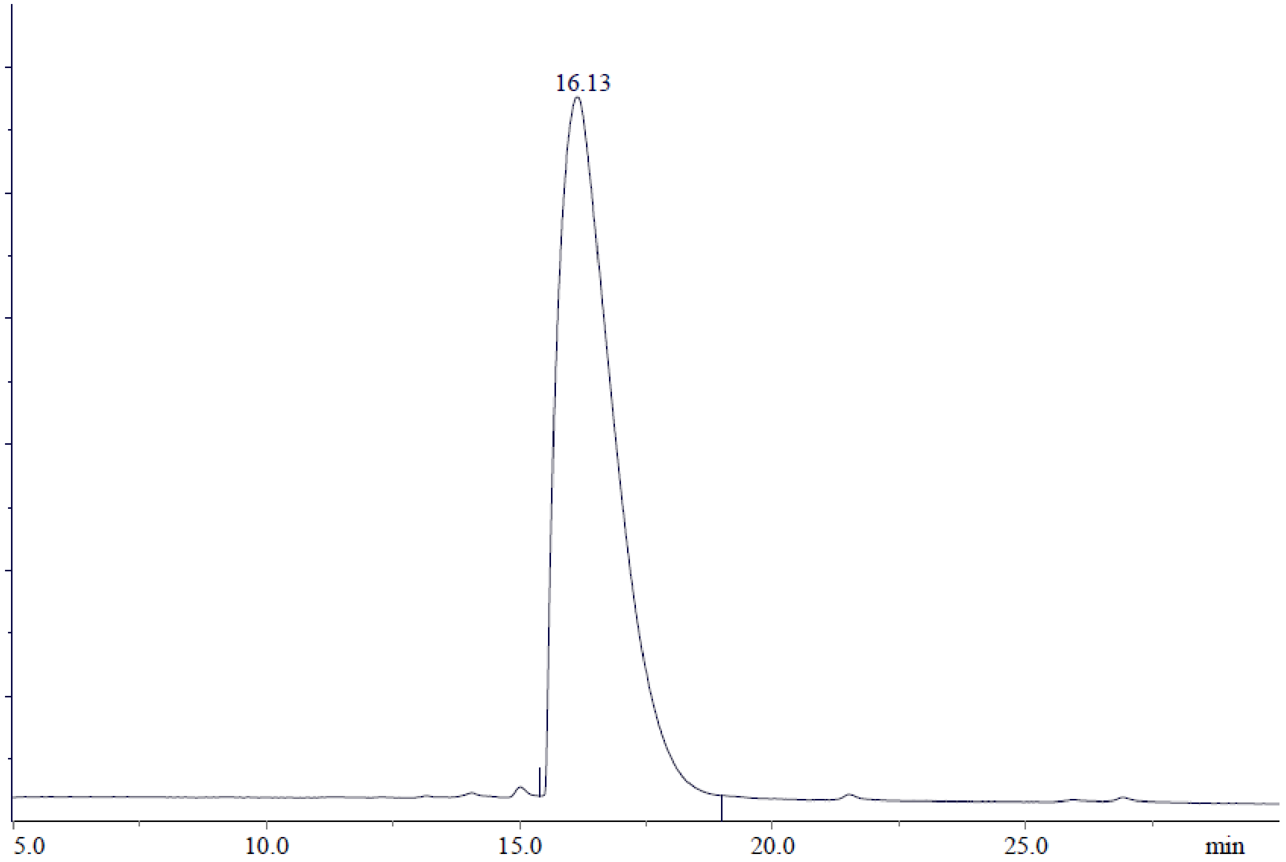

HPLC profile obtained for peptide **8** ([Api[8]]-Tric) (gradient: 45→75% B [CH_3CN/H_2O 9:1 + 0.05% TFA] in 30 minutes; flow rate: 1mL/min; C_{18} Phenomenex 300Å); R_t = 16.13 min.

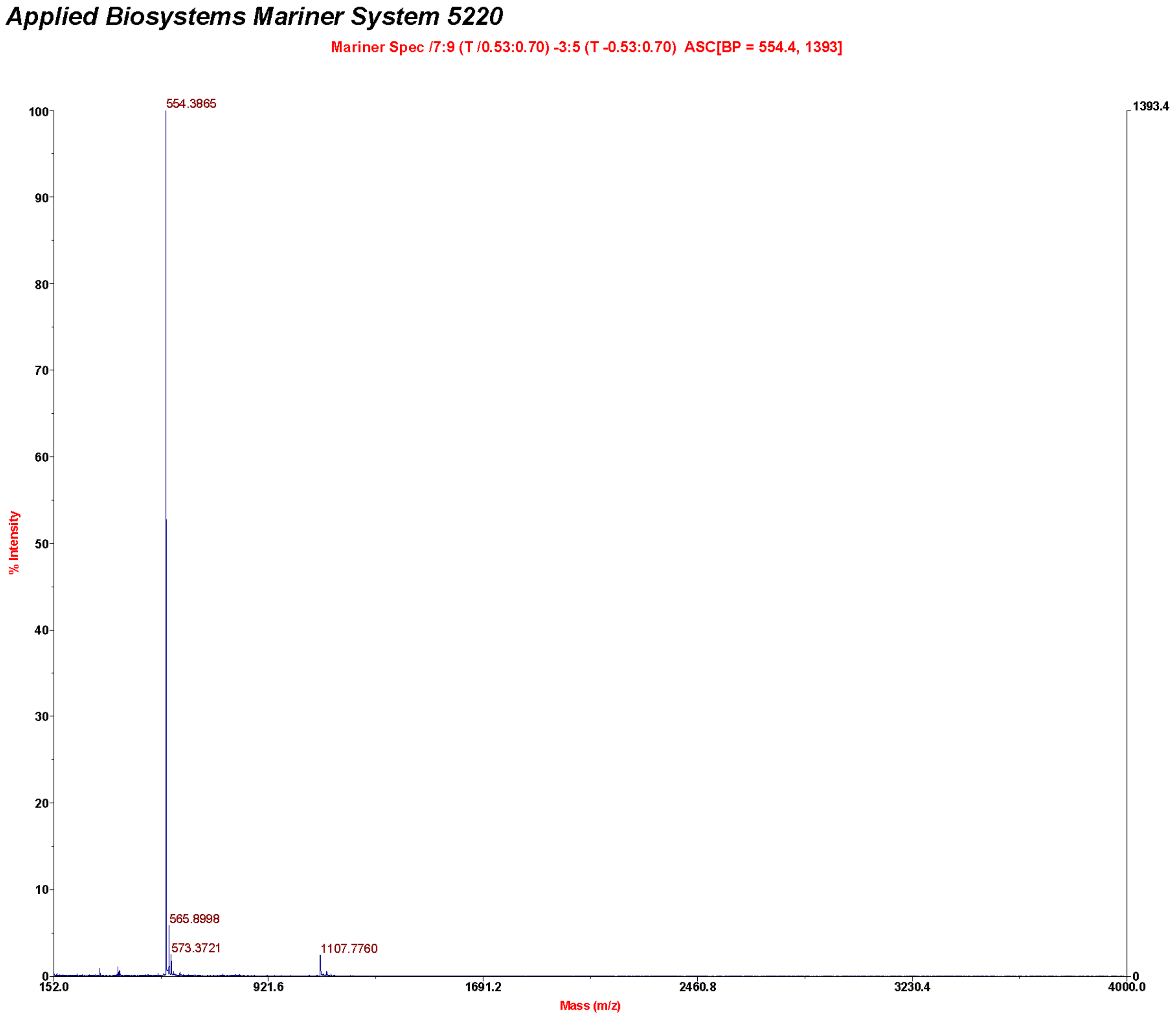

ESI-MS for peptide **8**: $[M+H]_{calcd.}$ 1107.7427; $[M+H]_{found}$: 1107.7760, $[M+2H^{2+}]_{found}$: 554.3865.

^{1}H NMR characterizations

peptide **1**   (**K25**)

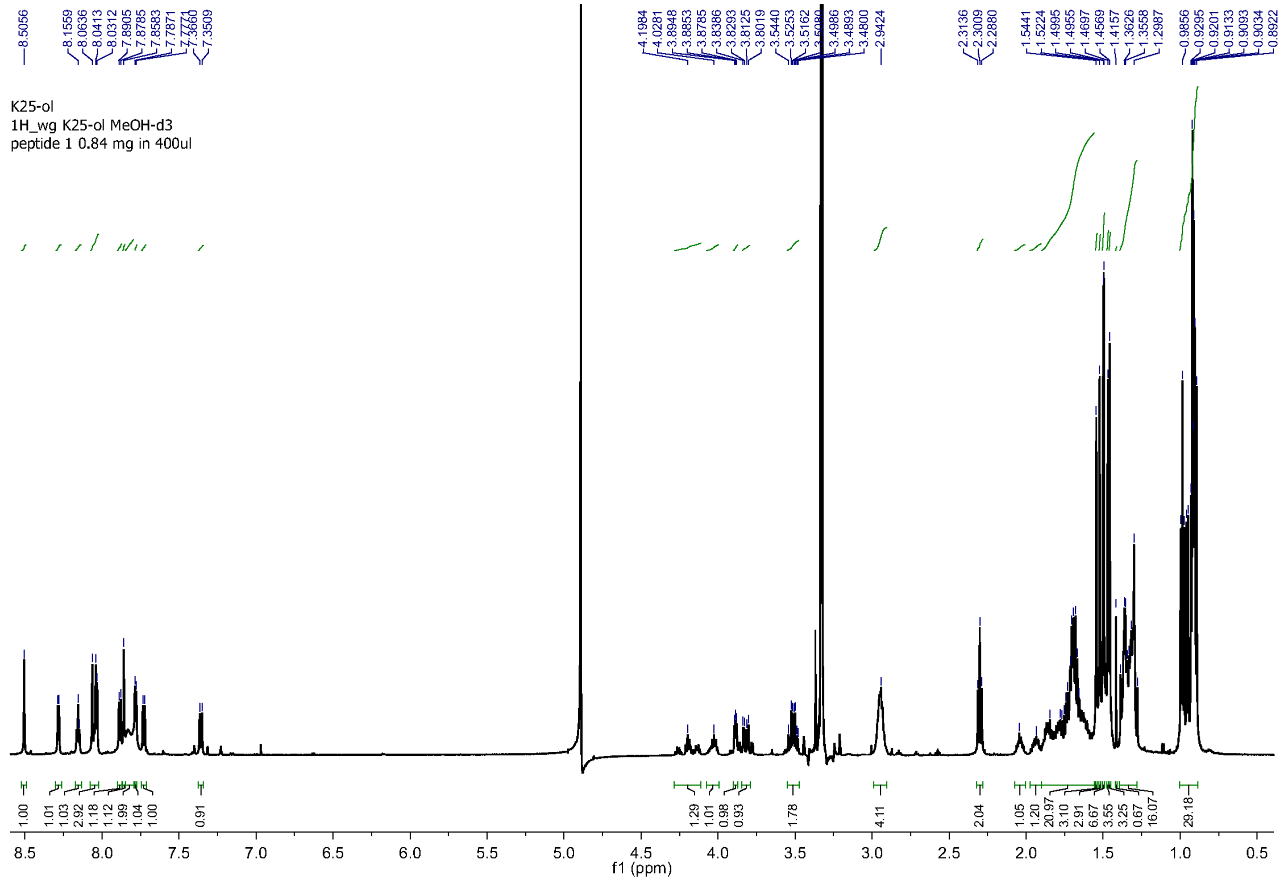

1D ^{1}H NMR spectrum for peptide **1** in CD_3OH, 298K, 600MHz (peptide concentration ca. 2 mM).

peptide **2** (**K2**)

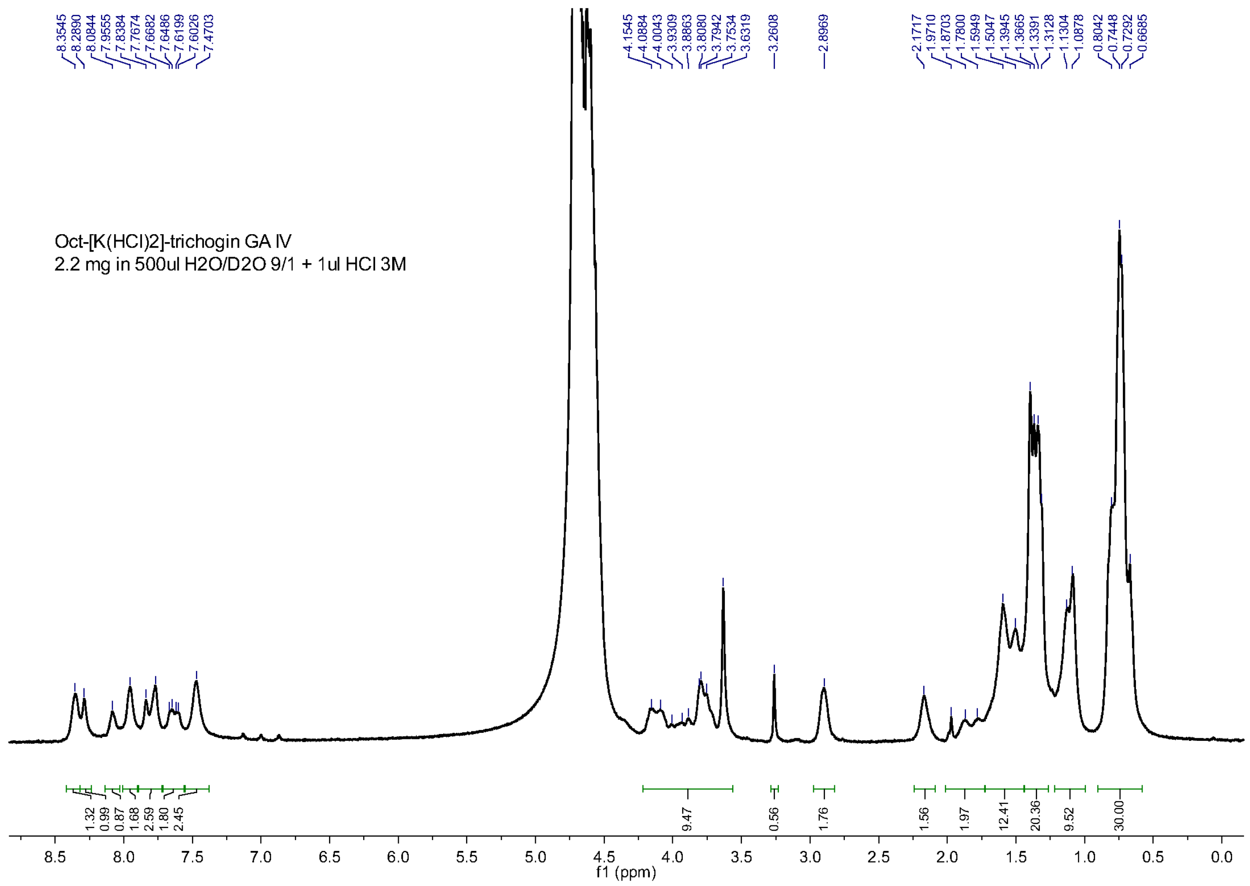

1D ^{1}H NMR spectrum for peptide **2** in H_2O/D_2O 9:1(+ 1µl HCl 3 M), 298K, 400MHz (peptide concentration ca. 4 mM).

peptide **3** (**K5**)

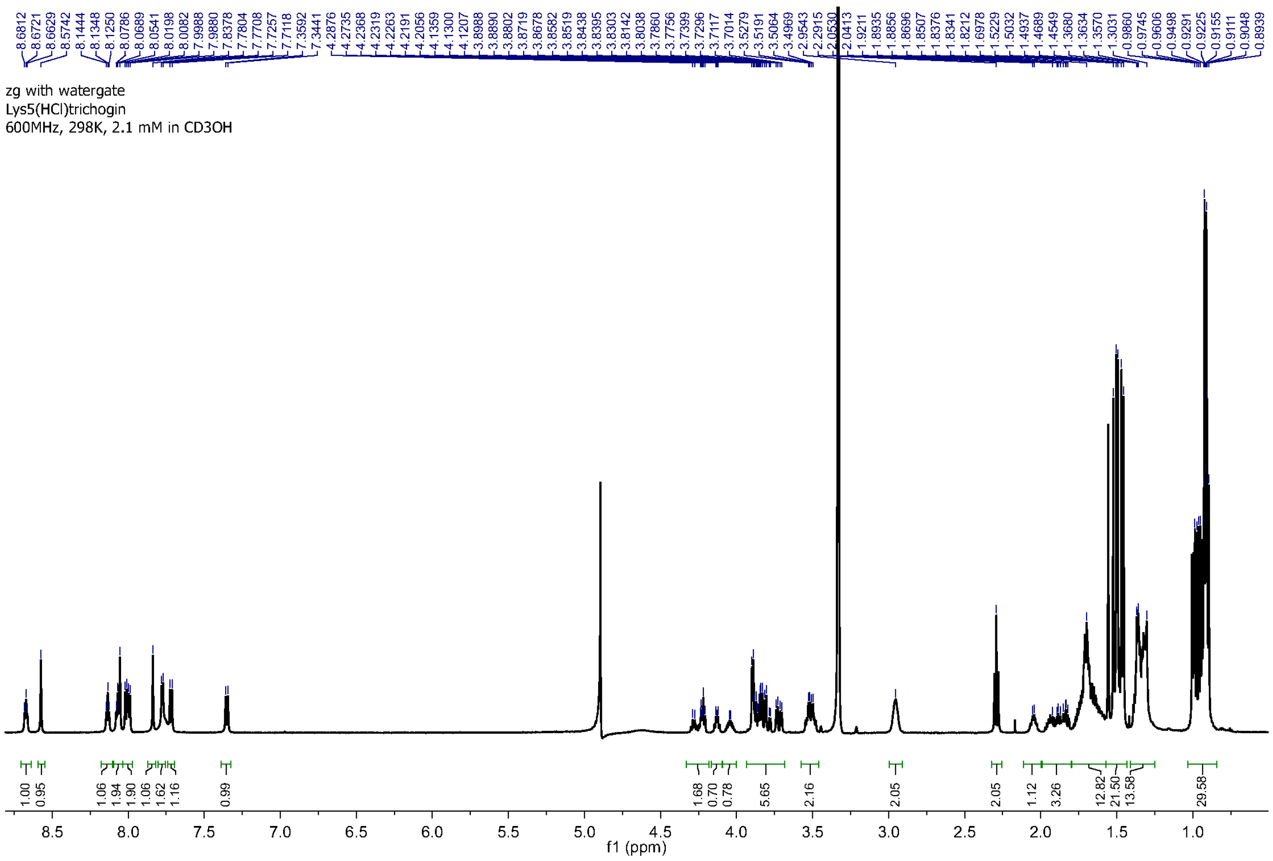

1D ^{1}H NMR spectrum for peptide **3** in CD_3OH, 298 K, 600MHz (peptide concentration ca. 2.1 mM).

peptide **4** (**K56**)

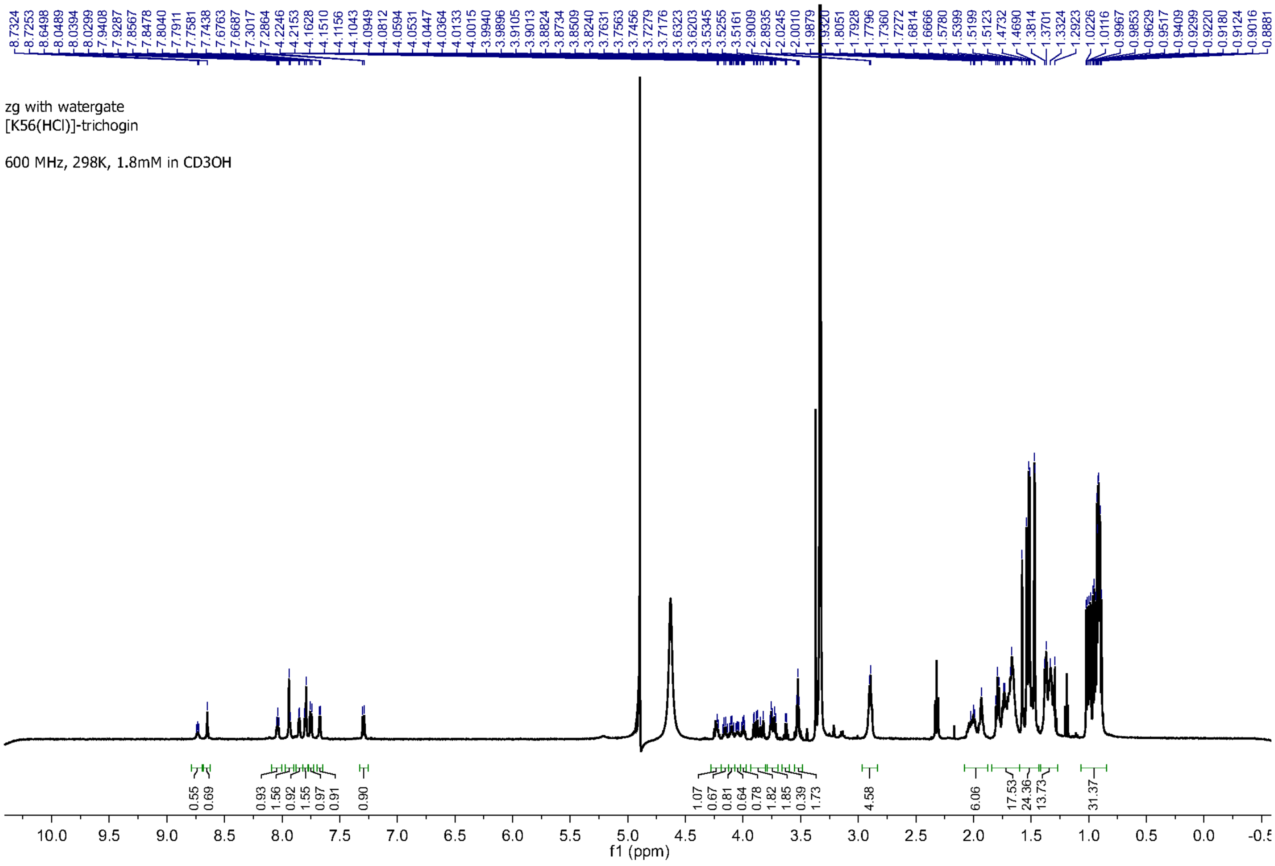

1D ^{1}H NMR spectrum for peptide **4** in CD_3OH, 298K, 600MHz (peptide concentration ca. 1.8 mM).

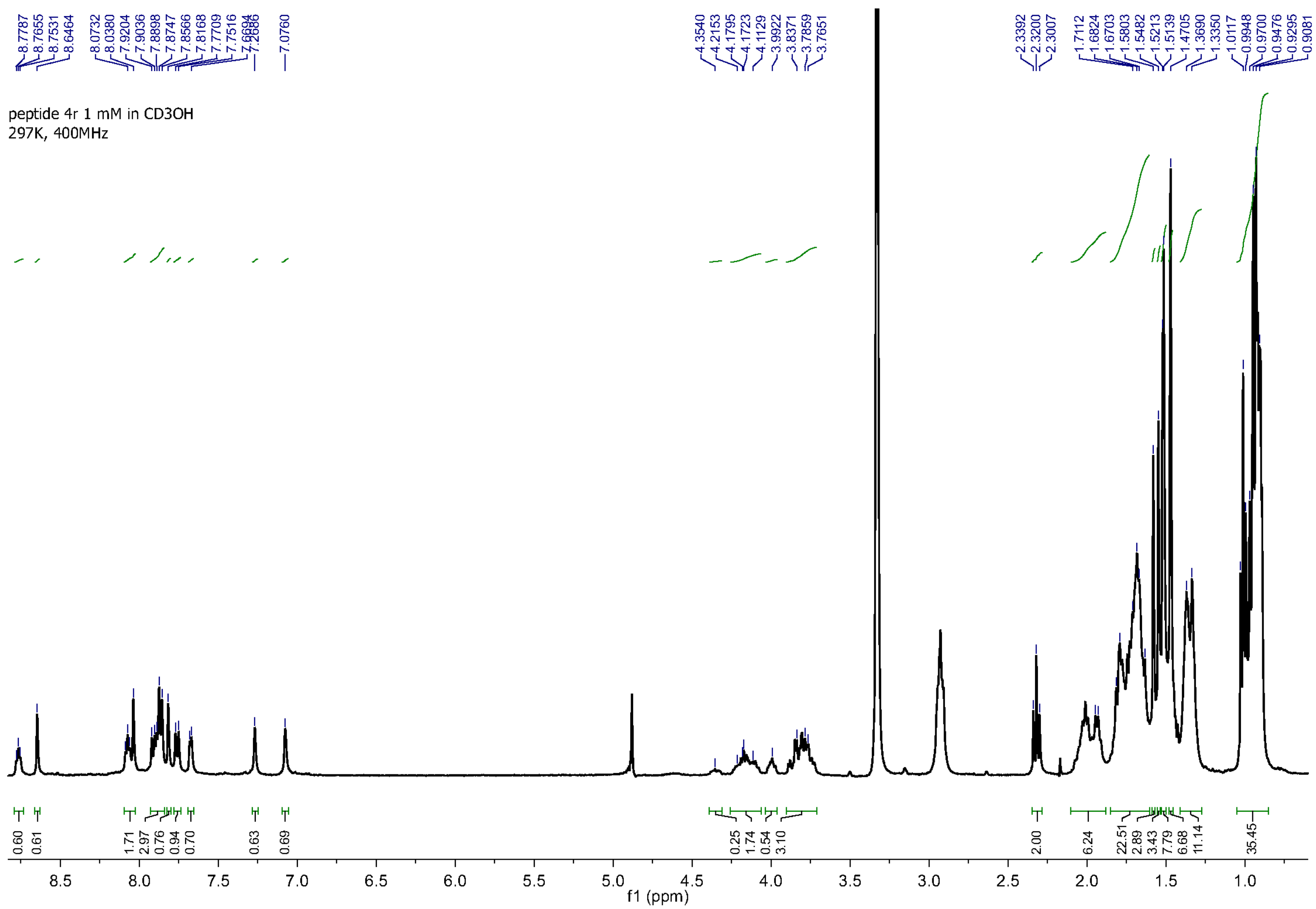

1D ^{1}H NMR spectrum for peptide **4r** in CD_3OH, 297K, 400MHz (peptide concentration 1 mM).

peptide **4c**

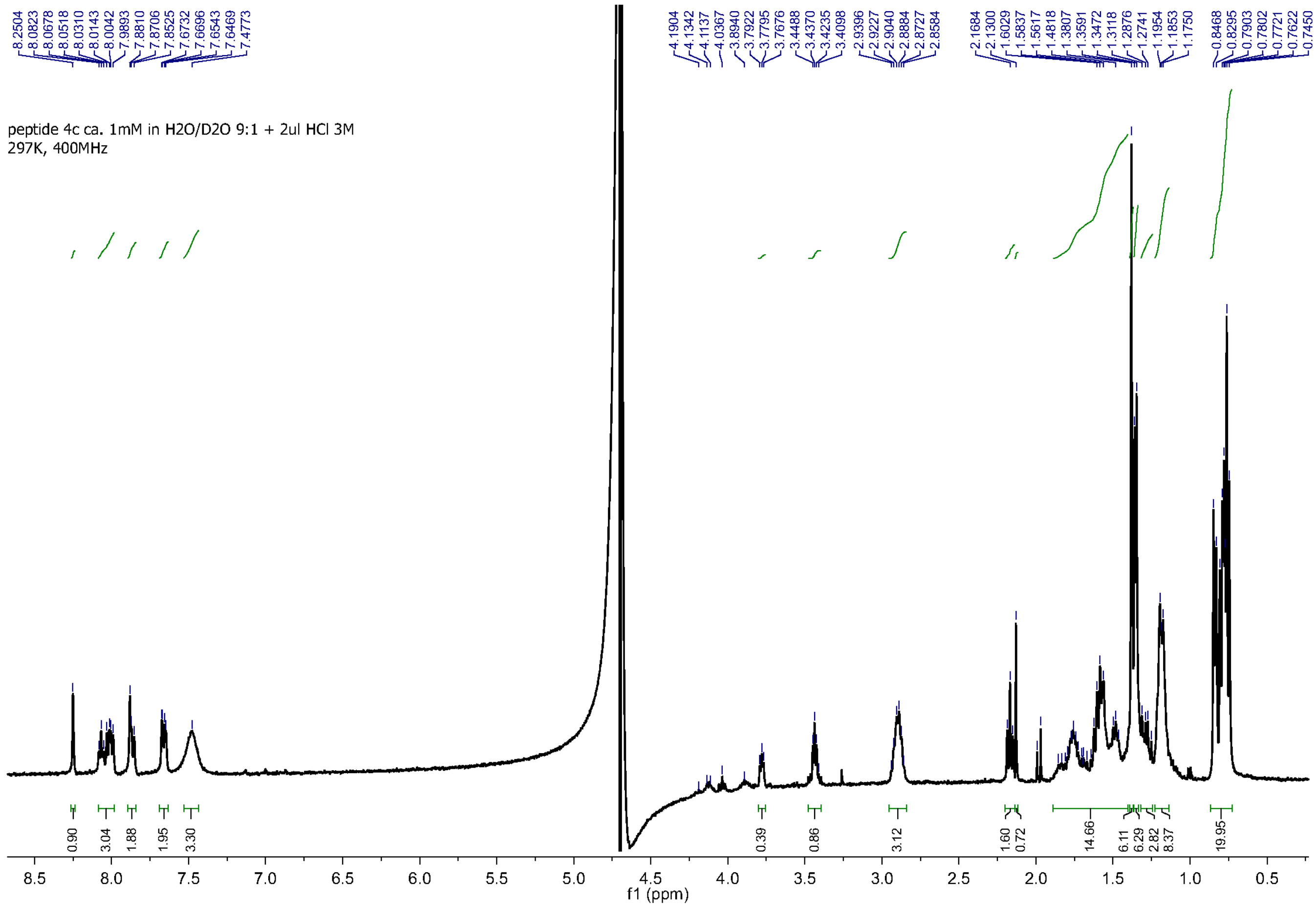

1D ^{1}H NMR spectrum for peptide **4c** in H_2O/D_2O 9:1 (+2µl HCl 3M in D_2O), 297K, 400MHz (peptide concentration 1 mM).

peptide **4c1**

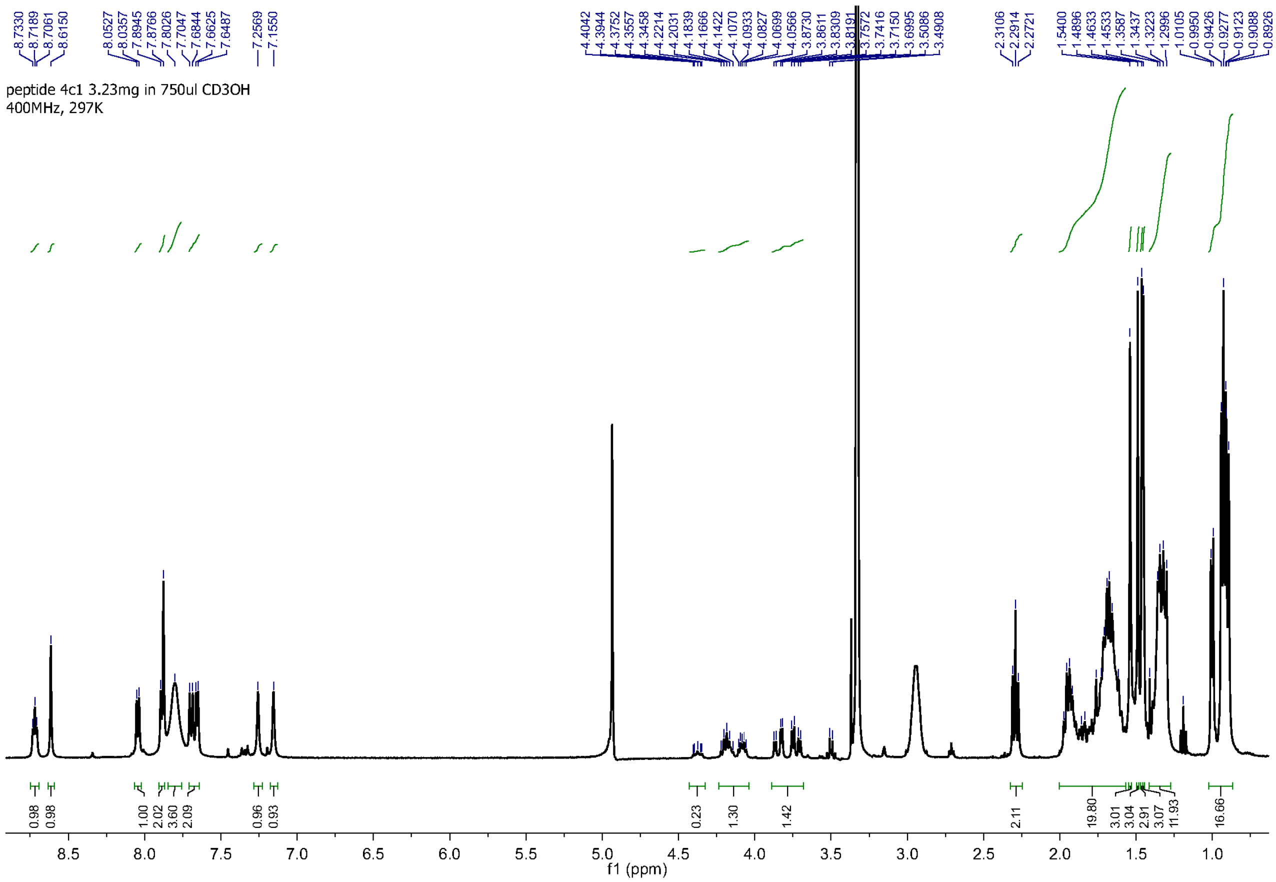

1D ^{1}H NMR spectrum for peptide **4c1** in CD_3OH, 297K, 400MHz (peptide concentration ca. 5 mM).

peptide **4c2**

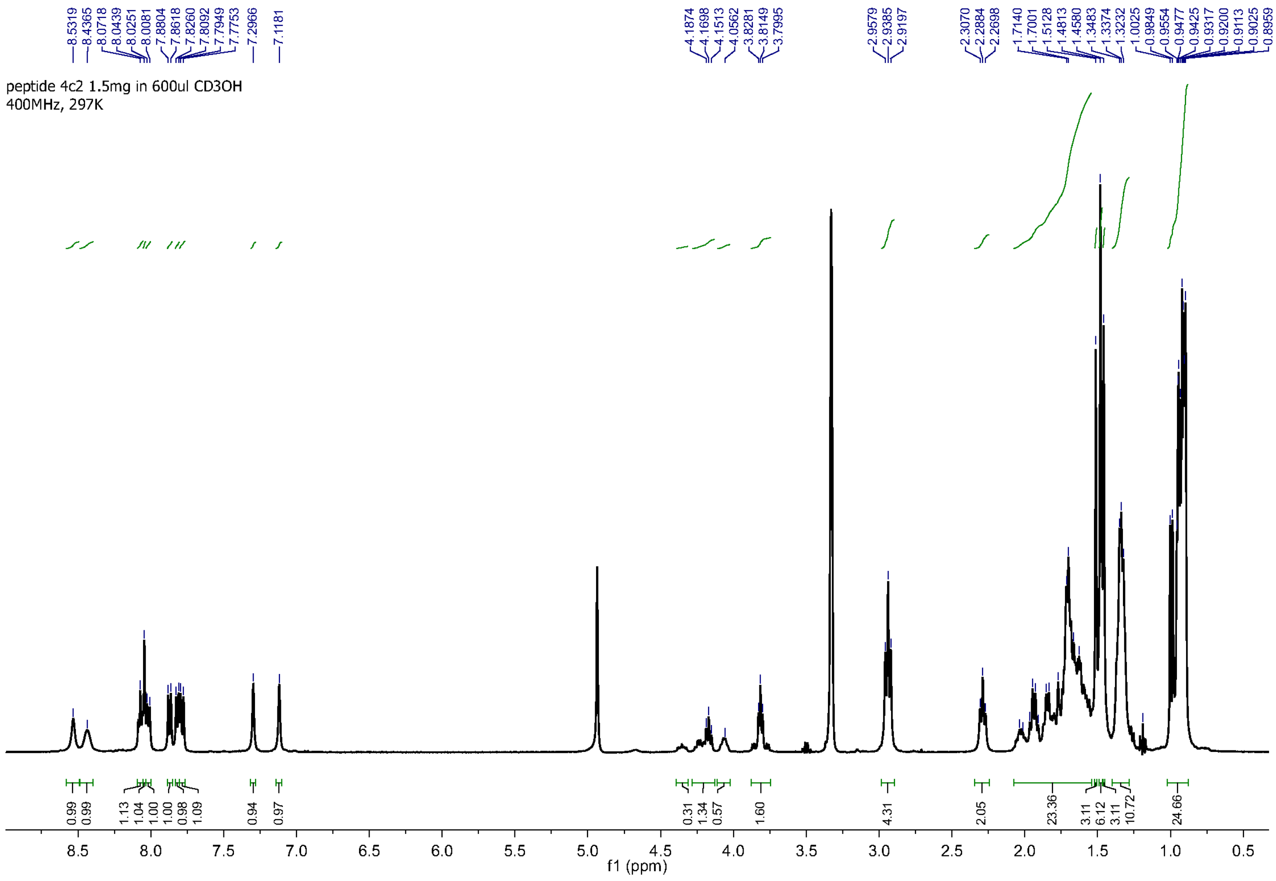

1D ^{1}H NMR spectrum for peptide **4c2** in CD_3OH, 297K, 400MHz (peptide concentration ca. 2.6 mM).

2D-NMR peptide **4c**

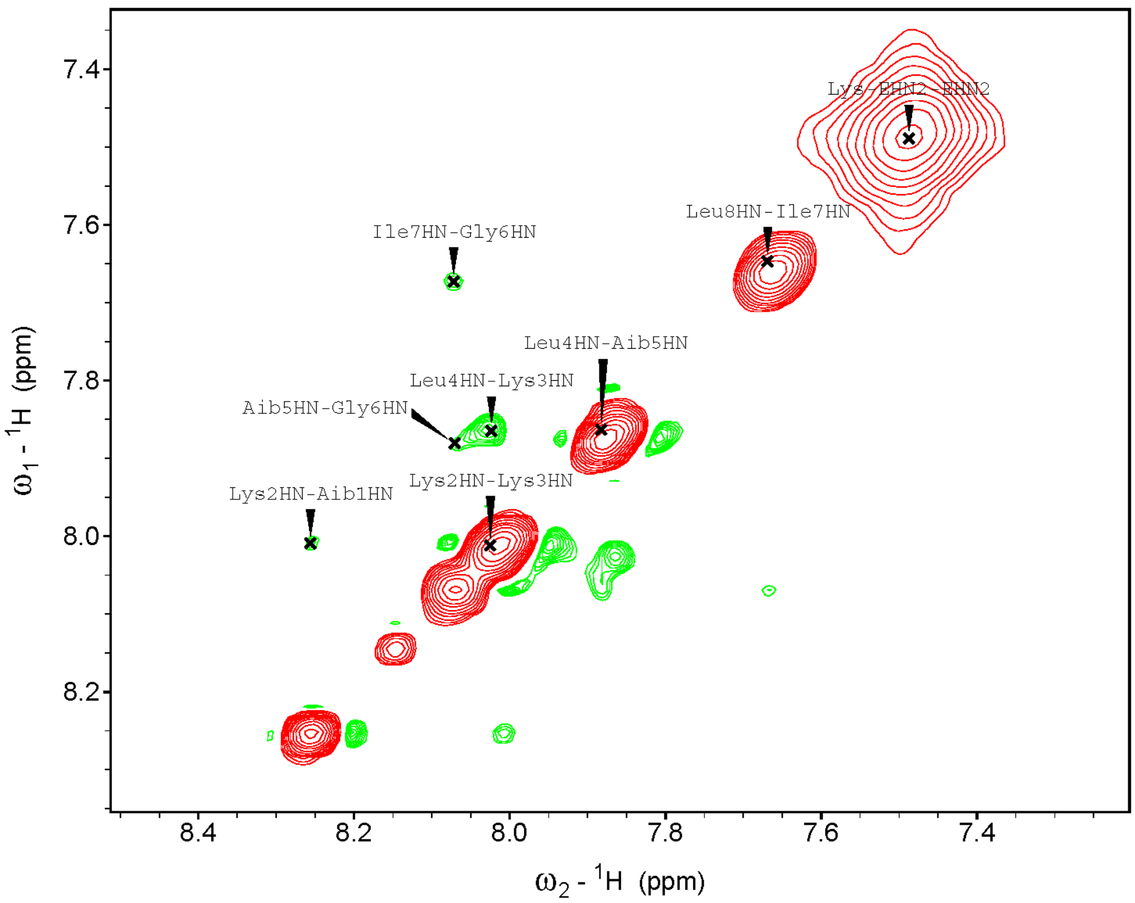

2D ^{1}H NMR for peptide **4c** in H_2O/D_2O 9:1 (+3μl HCl 3M in D_2O), 297K, 400MHz (peptide concentration 1 mM). NH-NH (amide) cross-peaks region of the ROESY spectrum. All sequential $NH_i \rightarrow NH_{i+1}$ are detectable (apart from those falling under the diagonal).

2D-NMR peptide **4c**

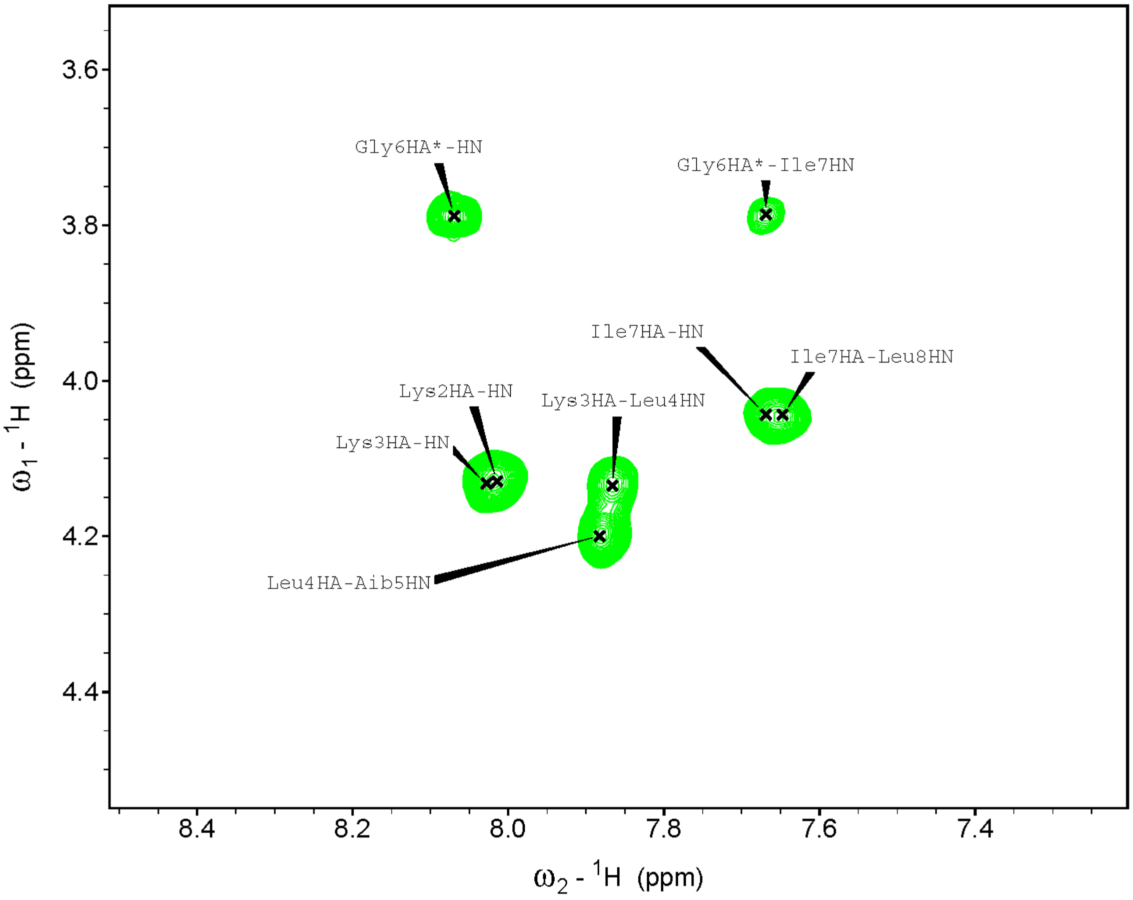

2D ^{1}H NMR for peptide **4c** in H_2O/D_2O 9:1 (+3µl HCl 3M in D_2O), 297K, 400MHz (peptide concentration 1 mM). αH-NH cross-peaks region (*fingerprint region*) of the ROESY spectrum. All sequential $\alpha H_i \rightarrow NH_{i+1}$ are detectable. There are no long-range correlations ($\alpha H_i \rightarrow NH_{i+n}$). This observation is (mainly) due to the extensive peak overlap, but it is usually associated with a somewhat flexible helical conformation, in agreement with CD findings.

2D-NMR peptide **4c1**

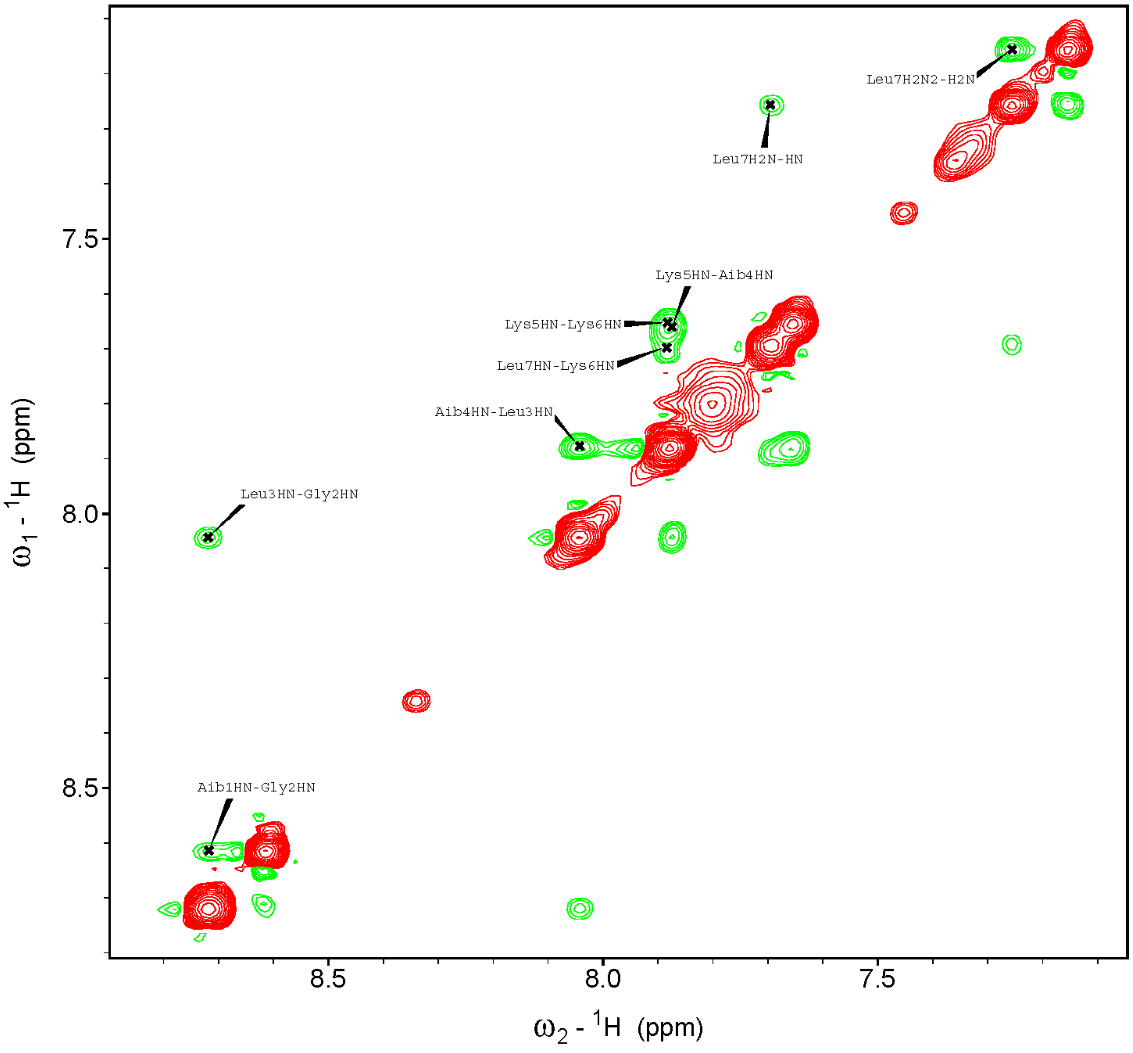

2D ^{1}H NMR for peptide **4c1** in CD_3OH, 297K, 400MHz (peptide concentration about 5 mM). The figure reports the NH-NH (amide) portion of the ROESY spectrum. All sequential $NH_i \rightarrow NH_{i+1}$ are detectable.

peptide **5** (**K6**)

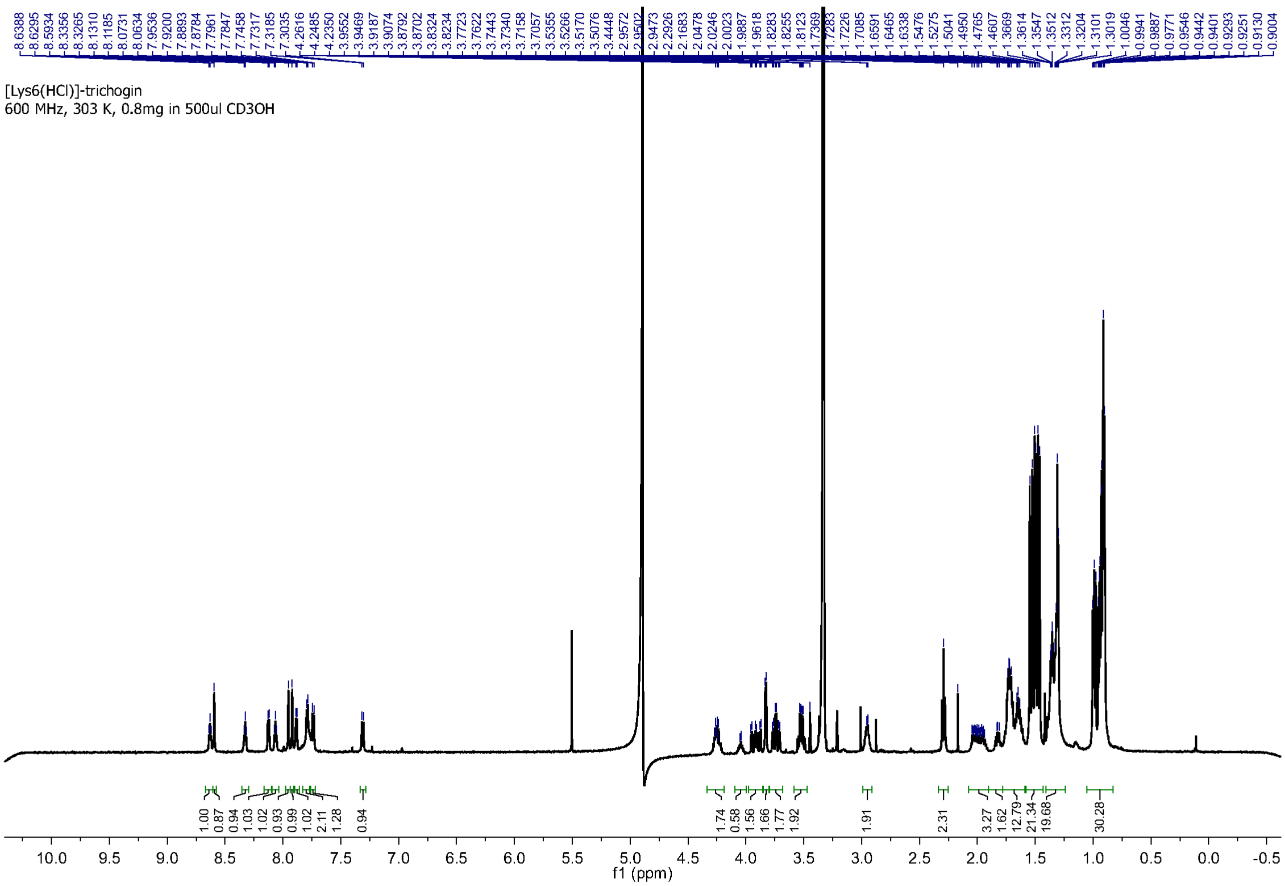

1D ^{1}H NMR spectrum for peptide **5** in CD_3OH, 298K, 600MHz (peptide concentration ca. 1.3 mM).

peptide **6** (**K5U6**)

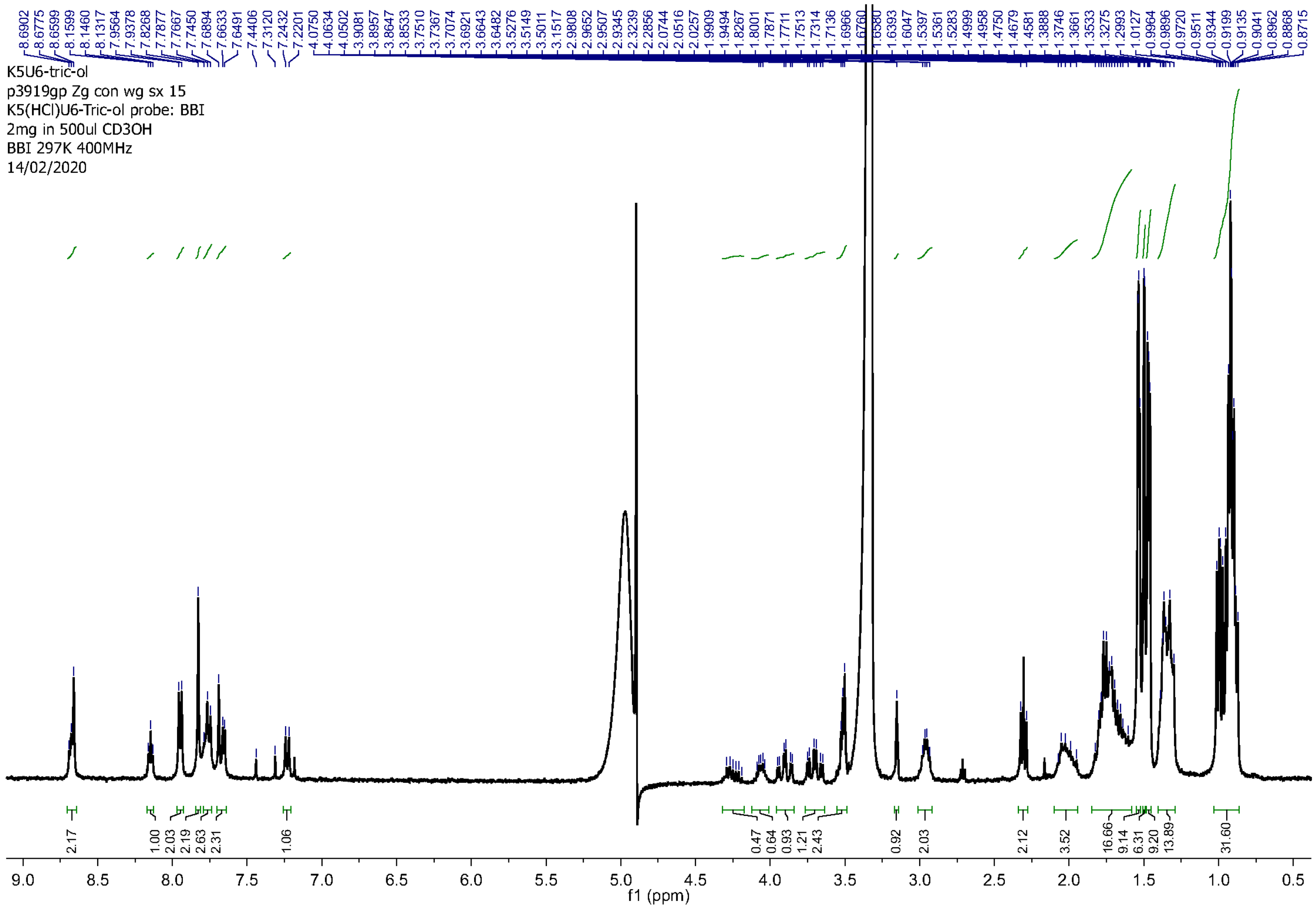

1D ^{1}H NMR spectrum for peptide **6** in CD_3OH, 297K, 400MHz (peptide concentration ca. 3 mM).

peptide **7** (**K26**)

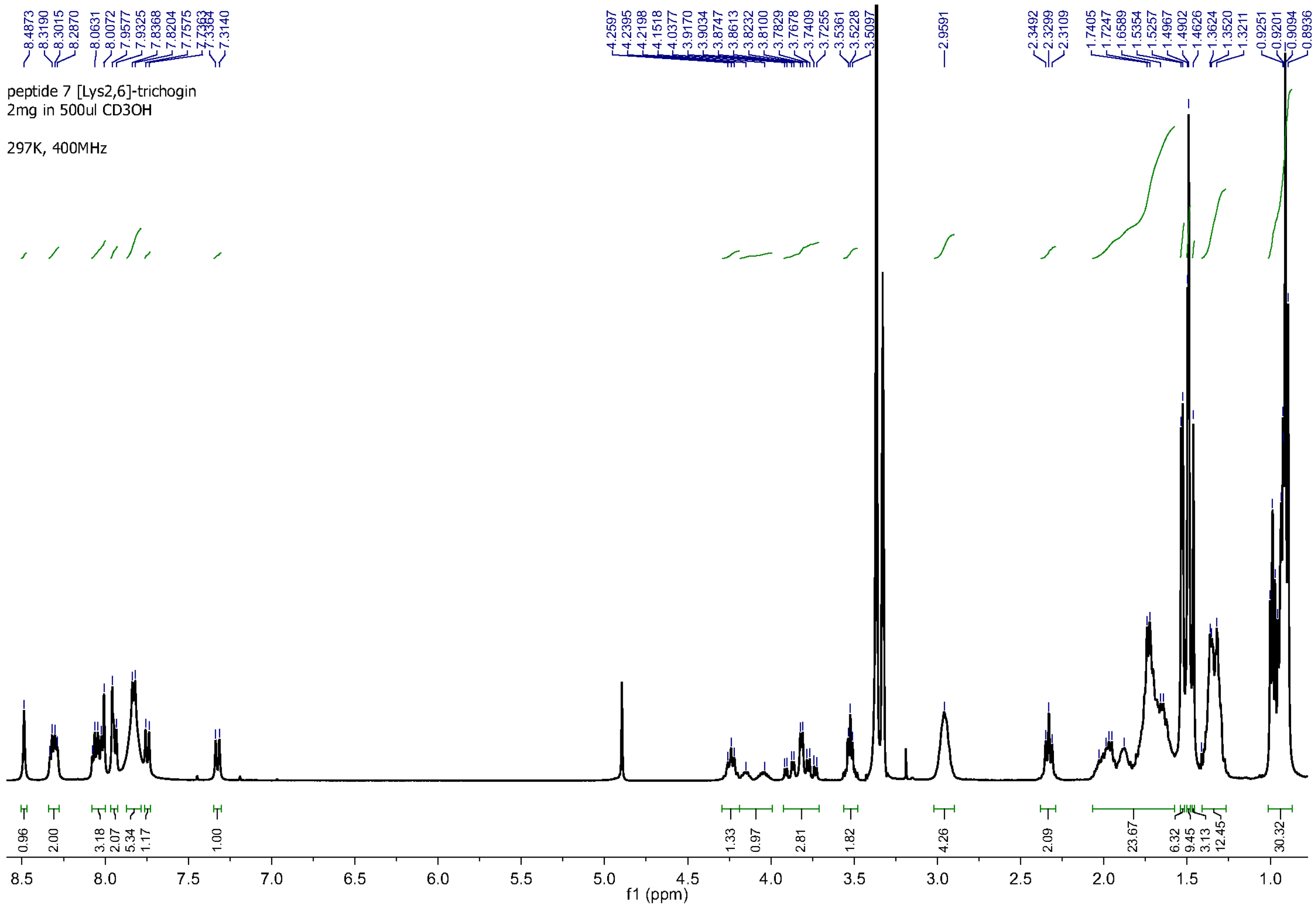

1D ^{1}H NMR spectrum for peptide **7** in CD_3OH, 297K, 400MHz (peptide concentration ca. 3 mM).

peptide **8** (**Api8**)

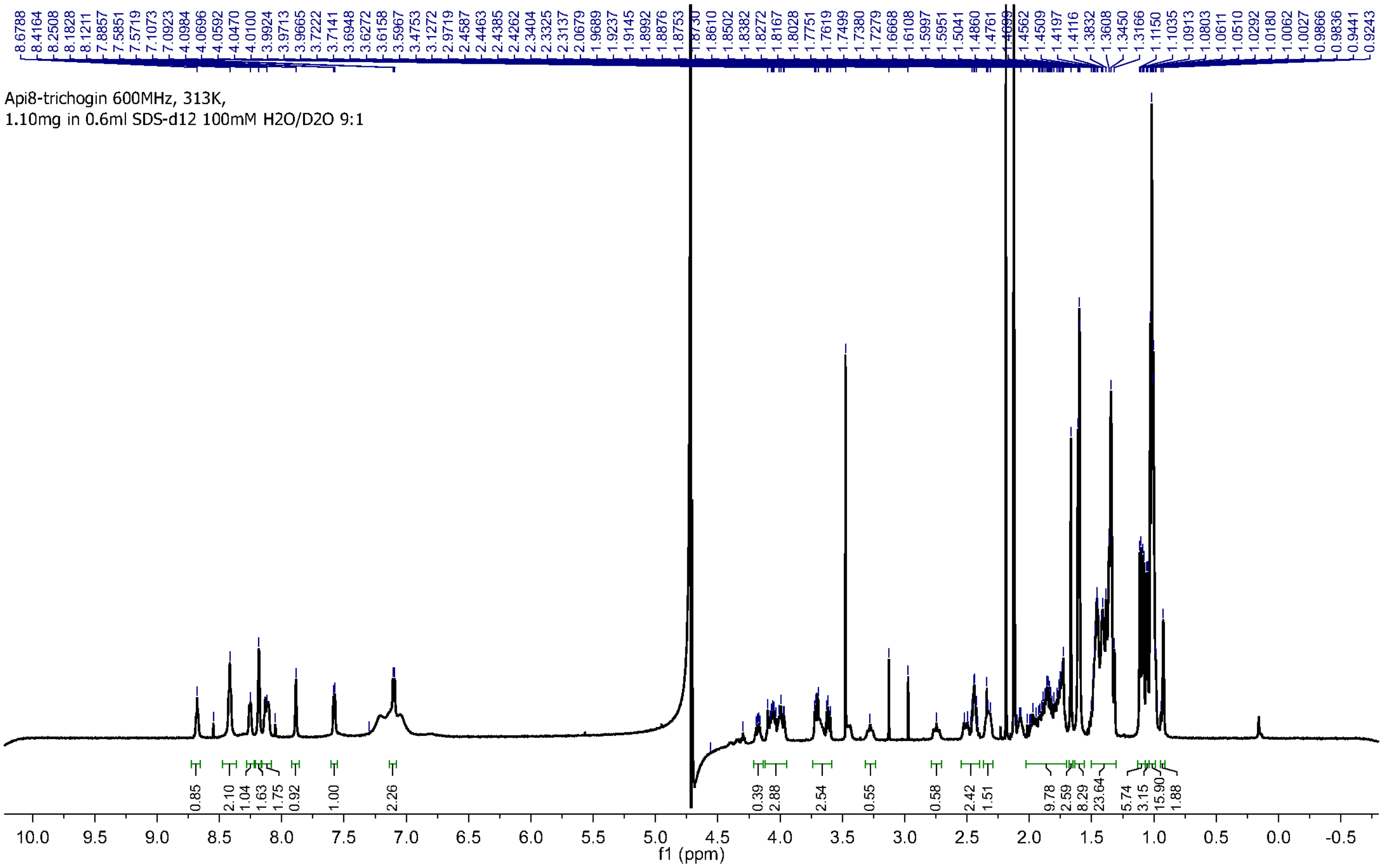

1D ^{1}H NMR spectrum for peptide **8** in SDS-d_{12} 100mM in H_2O/D_2O 9:1, 313K, 600MHz (peptide concentration ca. 2 mM).
